# Marine *Streptomyces*-Derived Novel Alkaloids Discovered in the Past Decade

**DOI:** 10.3390/md22010051

**Published:** 2024-01-22

**Authors:** Zijun Liu, Wenyan Sun, Zhe Hu, Wei Wang, Huawei Zhang

**Affiliations:** School of Pharmaceutical Sciences, Zhejiang University of Technology, Hangzhou 310014, China; 17857693513@163.com (Z.L.); 202005140518@zjut.edu.cn (W.S.); 18098450998@163.com (Z.H.); 2112107012@zjut.edu.cn (W.W.)

**Keywords:** marine *Streptomyces*, alkaloid, indole, pyrrole, pyridine, amide, anti-microbial effect, anti-inflammation, cytotoxicity

## Abstract

Natural alkaloids originating from actinomycetes and synthetic derivatives have always been among the important suppliers of small-molecule drugs. Among their biological sources, *Streptomyces* is the highest and most extensively researched genus. Marine-derived *Streptomyces* strains harbor unconventional metabolic pathways and have been demonstrated to be efficient producers of biologically active alkaloids; more than 60% of these compounds exhibit valuable activity such as antibacterial, antitumor, anti-inflammatory activities. This review comprehensively summarizes novel alkaloids produced by marine *Streptomyces* discovered in the past decade, focusing on their structural features, biological activity, and pharmacological mechanisms. Future perspectives on the discovery and development of novel alkaloids from marine *Streptomyces* are also provided.

## 1. Introduction

**Figure 1:**
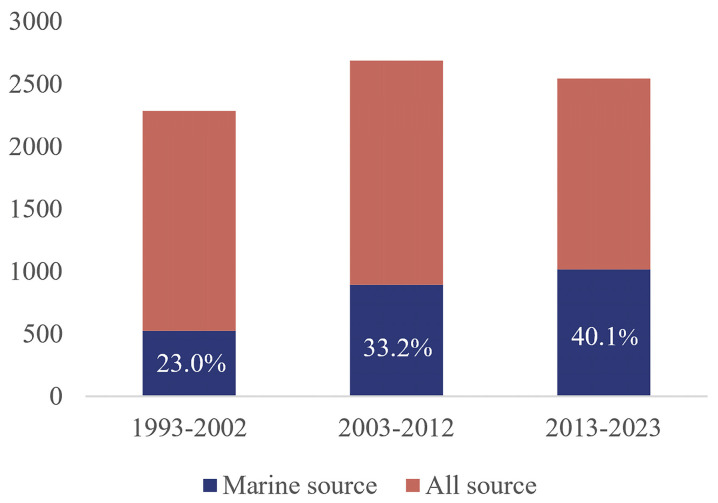
Source and statistics of new compounds from *Streptomyces* over the past three decades.

Extensive marine habitats differ greatly from the land in terms of temperature, pressure and inorganic salt content, thereby providing a wealth of ecological and biogenetic diversity [1]. Benefiting from technological advances in deep-sea resource extraction and microbial culture methods, the number of new microbial species with unique metabolisms has constantly expanded in recent years [2]. Natural products of marine microbial origin are more likely to have novel skeletons and significant pharmacological activity [3]. It is estimated that at least 30,000 compounds with therapeutic potential have been isolated from marine microorganisms; some of these substances have been used as lead compounds or biomaterials in new therapies such as drug-resistant cancer treatment [4,5]. Actinomycetes are one of the largest phyla of bacterial groups and are ubiquitous in both terrestrial and marine ecosystems [6]. Their biosynthetic gene clusters (BGCs) have well-known abilities in the metabolization of complex natural products [7]. *Streptomyces*, as the largest and most advanced genus of actinomycetes, is the source of 60% of natural antibiotics, and classic examples widely are used in clinical practice, including erythromycin, streptomycin and rifamycin [8,9]. In the past thirty years, the number of novel metabolites produced with marine *Streptomyces* as a percentage of the total source has increased from 23.0% to 40.1% per decade (Figure 1), suggesting that these *Streptomyces* strains play an increasingly important role in the production of new natural products.

**Figure 2:**
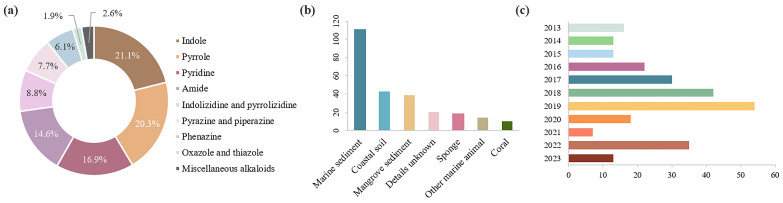
(**a**) Structural classes of marine *Streptomyces*-derived alkaloids reported in January 2013–June 2023; (**b**) sources of marine *Streptomyces* strains; and (**c**) number of new alkaloids discovered between January 2013–June 2023.

Alkaloids are the main chemical constituents in the secondary metabolites (SMs) of actinomycetes, and one of the compounds known to have the highest degree of druggability [10]. Most of these nitrogen-containing molecules have complex ring structures with promising pharmacological activity [11]. It has been demonstrated that marine alkaloids have widely clinical application value in the treatment of cancer, microbial infection, cardiovascular disease, inflammation, etc [12]. With the development of microbial genomics and metabonomics, the biosynthetic potential of marine *Streptomyces* has been deeply explored in the past decade. This review first provides a comprehensive overview of all new alkaloids produced by marine *Streptomyces* strains reported between January 2013 and June 2023. Three core databases (Web of Science, SciFinder and Dictionary of Natural Products) were used to search the targeted literature pertaining to specific topics related to marine and *Streptomyces* as well as novel or new alkaloids. According to their chemical structures, these metabolites (**1**–**261**) are grouped into nine types including indole, pyrrole, oxazole and thiazole, pyridine, pyrazine and piperazine, phenazine and phenoxazine, indolizidine and pyrrolizidine, amide and miscellaneous alkaloids. It is notable that indole, pyrrole, pyridine and amide are the major types, accounting for 72.9% of marine *Streptomyces*-derived novel alkaloids (Figure 2a). Detailed information for these substances is summarized in Table S1. In addition, *Streptomyces* strains isolated from marine sediments have a numerical advantage (Figure 2b); the number of new alkaloids produced by marine *Streptomyces* had a short-lived upward trend, but has fallen in the last two years (Figure 2c).

## 2. Indoles

Indoles are bicyclic alkaloids that usually use tryptophan or tryptamine as biosynthetic precursors [13]. They are common and grow rapidly in the SMs of marine-derived microorganisms with a wide range of biological activities [14]. Compounds **1**–**55** have been described as indole alkaloid derivatives of marine *Streptomyces* origin, including bisindole, indole sesquiterpenoid, and other miscellaneous indoles.

### 2.1. Bisindoles

Bisindole alkaloids represent a family formed by the oxidation and polymerization of two L-tryptophan molecules, which have more pronounced biological activities compared to the indole monomer structure [15]. These alkaloids have been reported to have cytotoxic, antibacterial, and antiviral activities; bisindole derivatives containing triazine groups have been used as targeted pyruvate dehydrogenase kinase (PDK) inhibitors in the treatment of pancreatic ductal adenocarcinoma [16,17]. This chapter highlights marine *Streptomyces*-derived staurosporine analogues and chlorinated bisindoles.

#### 2.1.1. Staurosporines

**Figure 3:**
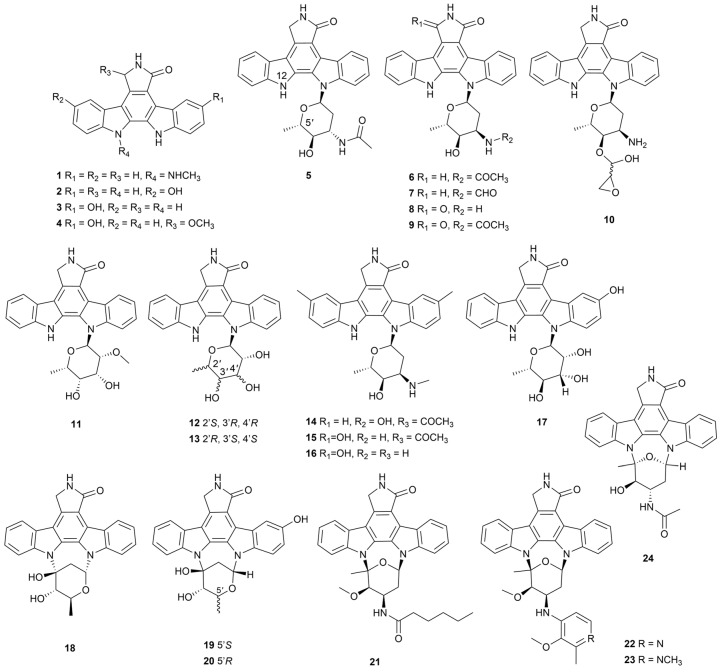
Chemical structures of staurosporine analogues **1**–**24** isolated from marine *Streptomyces*.

The potent protein kinase C (PKC) inhibitor (IC_50_ = 2.7 nM) staurosporine containing the indolo[2,3-*a*]carbazole structure was first discovered in *S. staurosporeus* in 1977 [18]. Lately, several analogues have been isolated and approved for clinical use, such as midostaurin and lestaurtinib [19]. Compounds **1**–**4** (Figure 3) are marine *Streptomyces*’ staurosporine derivatives with significant selective inhibition of Rho-associated protein kinase (ROCK2), PKC and Brution tyrosine kinase (BTK) [20,21]. A biosynthesis study indicated that the C-N bond linking the aglycone and deoxysugar moiety of staurosporine is catalyzed by cytochrome P450 enzymes [22]. Twelve holyrine A derivatives (**5**–**16,** Figure 3) displayed strongly or moderately cytotoxic and enzyme-inhibitory activity, with IC_50_ values ranging from 0.0057 to 16.6 µM [21,23,24,25]. When cultured in a liquid medium with 5-hydroxy-*L*-tryptophan precursors, strain *Streptomyces* sp. OUCMDZ-3118 was shown to produce another analogue 3-hydroxy-K252d (**17**, Figure 3), which demonstrated cytotoxicity against A549 and MCF-7 cell lines with IC_50_ values of 1.2 ± 0.05 µM, 1.6 ± 0.09 µM, respectively [26].

Moreover, streptocarbazoles C–H (**18**–**23**, Figure 3) were extracted from *Streptomyces* sp. DT-A65, DT-A61 and OUCMDZ-5380 [21,25,27]. Streptocarbazoles D and E rarely contained a hydroxyl group at the C-3 position. Compound **20** inhibited a PC3 cell line with an IC_50_ value of 5.6 μM, while compounds **21**–**23** inhibited acute myeloid leukemia cell line MV4-11 (IC_50_ = 0.81–1.88 µM). In addition, strain DT-A61 collected another staurosporine analogue (**24**, Figure 3), which exhibited extremely potent cytotoxic activity against PC3 cells with an IC_50_ value of 0.16 μM [21]. The structure–activity relationship (SAR) analysis showed that staurosporine analogs with a glycosyl unit double-linked to the aromatic aglycone by two C-N bonds displayed better biological effects.

#### 2.1.2. Halogenated Bisindoles

**Figure 4:**
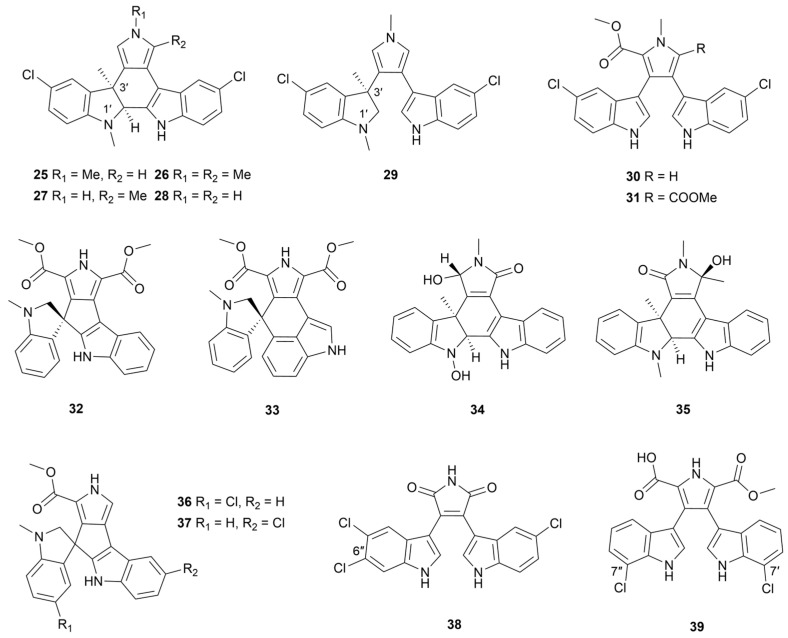
Chemical structures of halogenated bisindoles **25**–**39** isolated from marine *Streptomyces*.

To the best of our knowledge, all bisindoles from marine *Streptomyces* are chlorinated (**25**–**39**, Figure 4). Indimicins A−E (**25**–**29**) and lynamicins F–G (**30** and **31**) were obtained from a deep-sea-derived *Streptomyces* sp. SCSIO 03032 by solid phase extraction with XAD-16 resin [28]. These compounds had unusual 1′,3′-dimethyl-2′-hydroindole structures. Only dimethyl-substituted indimicin B (**26**) was seen to have antitumor activity against the MCF-7 cell line with an IC_50_ value of 10.0 μM. In order to characterize the function of gene *spmH*, which was predicted to be an L-Trp 5-halogenase, the authors inactivated this gene in strain SCSIO 03032 and obtained four bisindoles without a halogen substituent named spiroindimicins G–H (**32**–**33**) and indimicins F–G (**34**–**35**) [29]. It was confirmed that *spmH* functioned as halogenase and acted in early biosynthesis using L-Trp as a substrate. Compounds **32** and **33** showed various degrees of cytotoxicity against four cancer cell lines (SF-268, MCF-7, HepG2 and A549) and the presence or absence of chlorine atoms had no significant effect on the cytotoxic activity.

Two non-typical bisindole spiroindimicins E and F (**36** and **37**) were purified from the metabolites of strain MP131-18 with cluster 36 being predicted to be the gene cluster responsible for bisindole biosynthesis [30]. Only compound **36** showed weak cytotoxic activity against T24 bladder cancer cells, while **36**–**37** had no antimicrobial activity due to this type of effect was proportional to the amount of halogenation. In addition, *Streptomyces* sp. SCSIO 11791 produced two chlorinated bisindoles (**38** and **39**), which displayed moderate cytotoxicity against four tumor cells with IC_50_ values ranging from 2.9 μM to 19.4 μM [31]. Compound **38** additionally exhibited cytotoxic activity against MDA-MB-231 and NCI-H460 cell lines as well as inhibition of Gram-positive bacteria. The structure–activity relationship suggested that the substitution of the C-6″ position by the chlorine atom was more beneficial to the biological activity.

### 2.2. Indole Sesquiterpenoids

**Figure 5:**
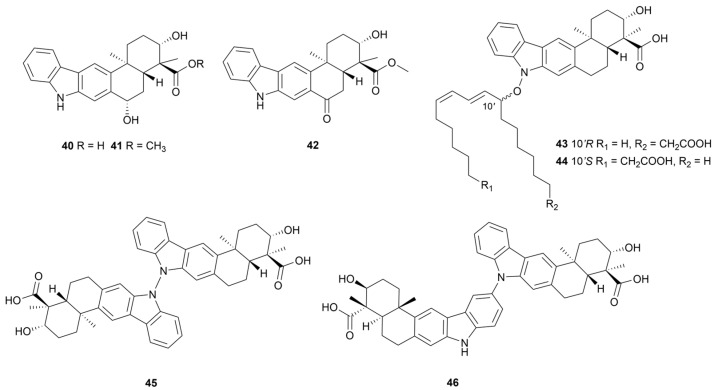
Chemical structures of Indole sesquiterpenoids **40**–**46** isolated from marine *Streptomyces*.

The first bacterial-derived indole sesquiterpenoid alkaloid, named xiamycin, was isolated from an endophytic *Streptomyces* sp. from *B. gymnorrhiza* in 2010 [32]. In recent years, compounds of this family have been found in marine *Streptomyces*, and have attracted attention because of their antibacterial, anti-human-immunodeficiency-virus (HIV) and anti-tumor activities [33]. Xiamycins C–E (**40**–**42**, Figure 5) were isolated from *Streptomyces* sp. HK-18 [34]. Compounds **41** and **42** exhibited strong activity against the replication of porcine epidemic diarrhea virus (PEDV) in a dose-dependent manner, with EC_50_ values of 0.93 μM and 2.89 μM, respectively. The mechanism of action inhibited the synthesis of key structural proteins for PEDV to prevent virus replication. The methyl ester group was an important functional group to maintain activity. Overexpression of the *orf2011* gene encoding the LuxR family regulator in the *Streptomyces* sp. HK-18 resulted in the production of two members of the xiamycins family containing an N-O bone linked aliphatic chain structure named lipoxiamycins A and B (**43** and **44**, Figure 5) as well as increased production of xiamycins dimers dixiamycins A and C (**45** and **46**, Figure 5) [35]. An anti-inflammatory assay showed that compounds **43** and **45** could significantly inhibit the production of lipopolysaccharide-induced NO with IC_50_ values of 9.89 ± 0.92 and 4.12 ± 0.22 μM, respectively.

### 2.3. Other Indoles

**Figure 6:**
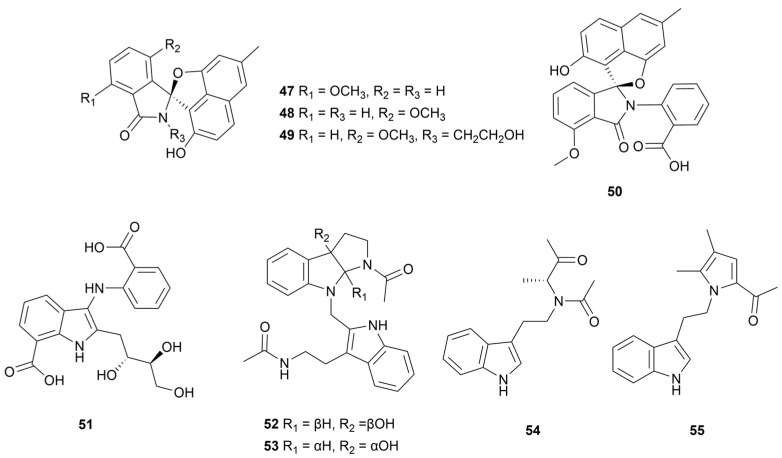
Chemical structures of other indoles **47**–**55** isolated from marine *Streptomyces*.

The first naturally derived indolinone-naphthofuran alkaloids, (±)-Pratensilins A–C (**47**–**49**, Figure 6), were isolated from *Streptomyces pratensis* KCB-132 obtained from a marine sediment from the Bohai Sea, China [36]. Only compound **47** displayed moderate cytotoxicity against eight human cancer cell lines (IC_50_ = 2.4 μM–67.4 μM), with small steric substituents in R_2_ and R_3_. After adding 50 µM of lanthanum chloride to the liquid medium, an indolinone-naphthofuran analogue (±)-Pratensilin D (**50**, Figure 4) was detected in the metabolites [37]. (-)-Pratensilin D (**50**) exhibited more potent biological activity against Gram-positive bacteria, yeast and five human cancer cell lines. One anthranilate-containing alkaloid, anthranosides C (**51**, Figure 6), was separated from a sponge-derived *Streptomyces* sp. CMN-62 (Naozhou Island, China) and exhibited a 171 μM IC_50_ against the influenza A H_1_N_1_ virus [38]. Four indole alkaloids streptoindoles A–D (**52**–**55,** Figure 6) were obtained from *Streptomyces* sp. ZZ1118 rice solid medium derived from a gut sample of marine shrimp (*Penaeus* sp.) [39]. Compound **54** potently inhibited *E. coli* and *C. albicans* (MIC = 7 μg/mL) and compound **55** was weakly active against methicillin-resistant *Staphylococcus aureus* (MRSA) only (MIC = 25 μg/mL). Compounds **52** and **53** were effective against all three pathogens, with MIC values of 7–25 μg/mL.

## 3. Pyrroles

Pyrrole alkaloids have more potential to be designed as lead compounds due to their property of easily forming hydrogen bonds [40]. Marine pyrrole derivatives usually have more unique structures and significant pharmacological activities such as antimicrobial, antiproliferative, anti-inflammatory and antiviral activities [41].

### 3.1. Pyrrolones and Pyrrolidones

**Figure 7:**
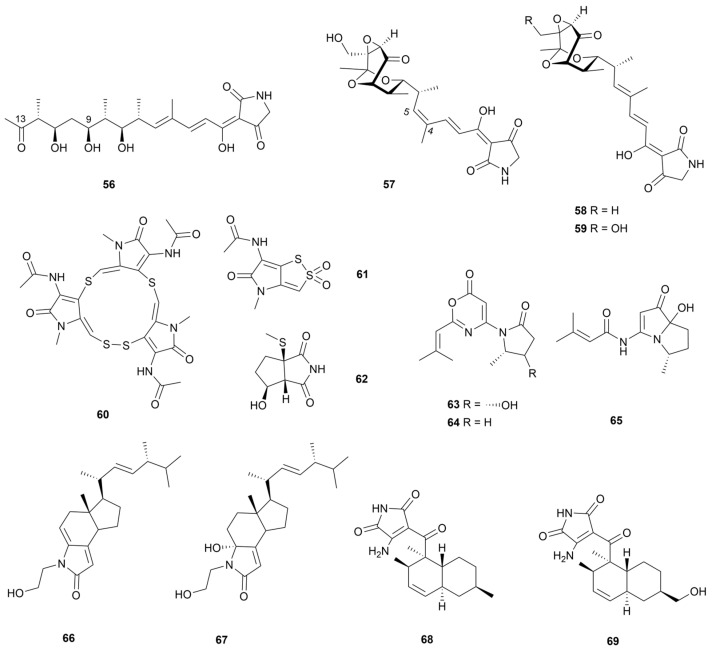
Chemical structures of pyrrolone and pyrrolidone alkaloids **56**–**69** isolated from marine *Streptomyces*.

This part describes cases in which pyrrole or pyrrolidine pentacyclic rings are replaced by carbonyl groups. The formed pyrrolone and pyrrolidone structures are crucial heterocyclic pharmacophores in medicinal chemistry, with significant biological activities [42]. The tirandamycins are a class of bacterial RNA polymerase (RNAP) inhibitors containing dienoyl tetramic acid and 2,4-pyrrolidinedione structures [43]. Two tirandamycin analogues tirandamycin K and isotirandamycin B (**56** and **57**, Figure 7), together with two known derivatives (**58** and **59**, Figure 7), were produced from marine *Streptomyces* strains [44,45]. Compound **56** was the first linear tirandamycin derivative that avoided bicyclic ketal ring formation due to the inability of the C-9*S* hydroxyl group to be nucleophilically attacked by C-13. In a pathogenic bacterial inhibitory activity assay, compounds **57**–**59** showed obvious antibacterial activity against *S. agalactiae* with MIC values of 2.5–5.0 μg/mL. However, compound **56** was inactive, therefore the authors deduced that the bicyclic ketal ring moiety was a necessary RNA polymerase target.

Three thio-containing pyrrolone-based alkaloids (**60**–**62,** Figure 7) were discovered from two marine *Streptomyces* [46,47]. Thiolopyrrolone A (**60**) had an unusual macrocyclic skeleton and also inhibited Bacille Calmette–Guérin (BCG), *M. tuberculosis*, and *S. aureus*, with MIC values of 10, 10 and 100 µg/mL, respectively. Bacillimide (**62**) had a rare cyclopenta[c]pyrrole-1,3-dione structure bearing a methylsulfide group. This pyrrolidone alkaloid was an isocitrate lyase (ICL) enzyme moderate inhibitor under C_2_-carbon-utilizing conditions, demonstrating an IC_50_ value of 44.24 µM against *C. albicans*. In addition, *Streptomyces* sp. KMF-004 yielded two unusual pyrrolidinyl-oxazinone alkaloids, Salinazinones A and B (**63** and **64**, Figure 7) [48]. Compound **64** showed an inhibitory effect on lipopolysaccharide (LPS)-induced NO production by BV-2 microglia cells (an IC_50_ value of 17.7 μM). The authors speculated that the oxazinone structure was synthesized by amide cyclization and gave a possible biosynthetic intermediate named bohemamine D (**65**). Anandins A and B (**66** and **67**, Figure 7), as two unique pyrrolidone-containing steroidal alkaloids, were isolated from *Streptomyces anandii* H41-59 derived from mangrove sediments [49]. The compound **66** showed moderate inhibitory effects against cancer cell lines MCF-7, SF-268 and NCI-H460 (IC_50_ = 7.5–7.9 µg/mL, respectively). Two pyrrolidone derivatives, ligiamycins A and B (**68** and **69**, Figure 7), were obtained by co-culture of two marine-derived strains *Streptomyces* sp. GET02.ST and *Achromobacter* sp. GET02.AC [50]. A biological evaluation showed that compound **68** exhibited moderate effects against *S. aureus* and *S. enterica* (both MIC values of 16 µg/mL), while compound **69** was cytotoxic against HCT116 cancer cells (IC_50_ = 20.1 µM). The presence of the hydroxyl group in compound **69** had an opposite effect on antitumor activity and antibacterial activity.

### 3.2. Pyrrolobenzodiazepines

**Figure 8:**
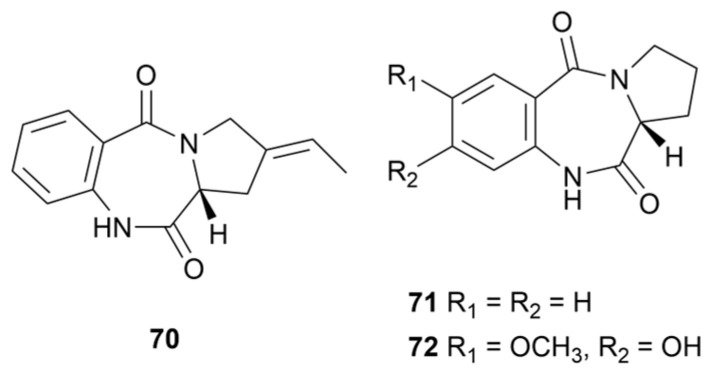
Chemical structures of pyrrolobenzodiazepines **70**–**72** isolated from marine *Streptomyces*.

Pyrrolo[1,4]benzodiazepines (PBDs, Figure 8) are tricyclic alkaloids that can be divided into [2,1-*c*][1,4], [1,2-*a*][1,4] and [1,2-*d*][1,4] structural types according to the different positions of the pyrrole ring-binding [51]. Natural PBDs are originally derived from *Streptomyces* as DNA alkylating antitumor drugs, and the classical representatives are anthramycin, sibiromycin and tomaymycin [52]. A pyrrolobenzodiazepine alkaloid, oxoprothracarcin (**70**), was produced by *Streptomyces* sp. M10946 obtained from a mangrove sediment [53]. Compound **70** had antiproliferative effects against MDA-MB-231 cells and A549 cells at 10 μM, with growth inhibition rates of 10.2% and 7.3%. An unusual PBD derivative, 7-methoxy-8-hydroxy cycloanthranilylproline (**71**), along with a known analogue (**72**), was isolated from marine *S. cacaoi* 14CM034 and displayed antimicrobial effects on *E. coli*, MRSA, *E. faecium*, *P. aeruginosa* and *C. albicans*, with MIC values ranging from 8.75 to 32 µg/mL [54]. It seems that methoxy substitution on the benzene ring of PBD diminished the antimicrobial effect.

### 3.3. Ansamycins

**Figure 9:**
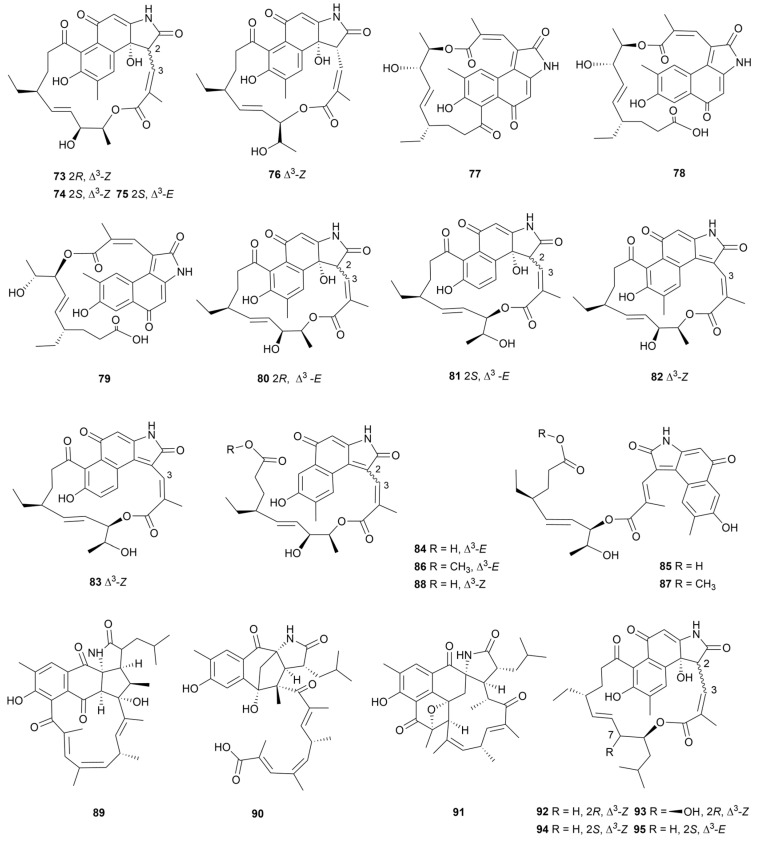
Chemical structures of ansamycin analogues **73**–**95** isolated from marine *Streptomyces*.

Ansamycins are a class of important macrocyclic lactam alkaloids obtained mainly from actinomycetes, of which the more representative include the anti-tuberculosis drug laofomycin, Hsp90 inhibitor geldamycin, and anticancer drug ansamitocin P-3 [55]. According to the different aromatic cores, ansamycins can be divided into benzene series and naphthalene series [56]. Hygrocins are a subclassification of naphthalenic ansamycins, whose amides are five-membered or seven-membered nitrogen heterocycles [57]. After knocking out the gene *gdmAI* responsible for the synthesis of the high-yield ansamycin analogue geldanamycin in *Streptomyces* sp. LZ35, hygrocins C−F (**73**–**76**, Figure 9) were produced and characterized [58]. In 2015, strain LZ35 was genetically modified by overexpression of *hgc1*, a LAL-type activator, and was found to produce three hygrocin derivatives, hygrocins H–J (**77**–**79**, Figure 9) [59]. Compounds **73**, **74**, **76** and **77** were shown to be cytotoxic to MDA-MB-231, PC3 and HeLa cell lines, with IC_50_ values of 0.5–5.0 μM. *Streptomyces* sp. ZZ1956 afforded nine derivatives named hygrocins K–S (**80**–**88**, Figure 9) [60]. Compounds **83**, **86** and **87** showed antiproliferative activity against human glioma U87MG and U251 cells with IC_50_ values of 7.04-10.46 μM. In addition, compounds **83**, **84**, and **87** displayed antibacterial activity against MRSA and *E. coli* (MIC = 8–24 µg/mL). The *E* configuration of C-3 and C-4 double bond and the existence of the *ansa* ring in these molecules are the keys to their strong biological activity.

Three ansamycin derivatives with unprecedented skeletons, ansalactams B–D (**89**–**91,** Figure 9), were isolated from marine sediment-derived *Streptomycetes* sp. CNH189 [61]. Antibacterial activity tests showed that compounds **89**–**91** had weak inhibitory activity against MRSA, with MIC values of 31.2, 31.2, and 62.5 μg/mL, respectively. A chemical study of the strain *Streptomyces* sp. KFD18 produced four ansamycin analogues named divergolides T–W (**92**–**95,** Figure 9) [62]. All these substances showed various degrees of cytotoxic activity against cancer cell lines SGC-7901, K562, Hela and A549; compounds with hydroxylation of C-7 and *R* configuration of C-2 exhibited weak cytotoxic effect.

### 3.4. Other Pyrroles

**Figure 10:**
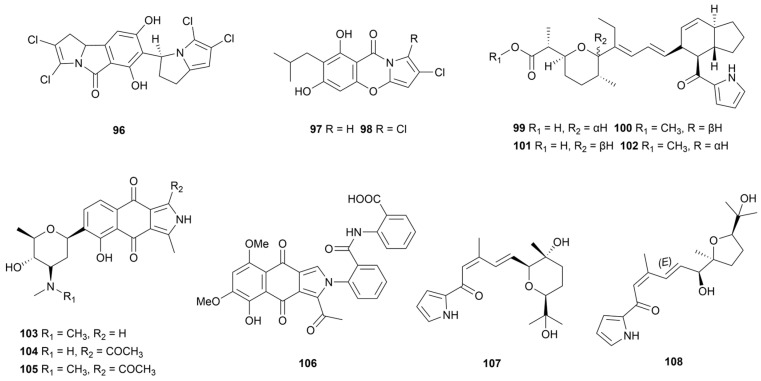
Chemical structures of other pyrroles **96**–**108** isolated from marine *Streptomyces*.

Chlorizidine A (**96**, Figure 10), biosynthesized based on the non-ribosomal peptide synthetases/polyketide synthases (NRPS/PKS) pathway, was an unprecedented alkaloid containing chlorinated 5H-pyrrolo[2,1-*a*]isoindol-5-one fragment [63]. It exhibited an IC_50_ value of 3.2–4.9 μM against the HCT-116 adenocarcinoma cell line with the pyrrole isoindolone ring moiety as a key pharmacophore. Isolated from marine sediment samples, the *S. zhaozhouensis* 208DD-064 produced two halogenated pyrroles, streptopyrroles B and C (**97** and **98**, Figure 10) [64]. Compounds **97** and **98** showed promising activity with three Gram-positive bacteria (MIC = 0.7–2.9 µM), with inverse correlation to the number of halogen substituents. Moreover, streptopyrrole B (**97**) exhibited moderate activity against six cancer cell lines. Three pyrrole ether compounds of the indanomycin family (**99**–**102**, Figure 10) were isolated from *S. antibioticus* PTZ0016 extracts based on antimicrobial activity [65]. A biological evaluation showed that compounds **99**–**102** inhibited the growth of *S. aureus* with MIC values of 4.0–8.0 µg/mL. Nitricquinomycins A–C (**103**–**105**, Figure 10) arethe first example of naphthopyrroledione derivatives containing an angolosamine moiety and **105** had a significantly inhibitory effect on A2780 cell lines (IC_50_ = 4.77 µM) and moderate activity against *E. coli*, *S. aureus* and *C. albicans* (MIC values of 20–40 µM) [66]. In 2021, a nitricquinomycin analogue, bhimamycin J (**106**), was purified from *Streptomyces* sp. MS180069 and demonstrated 79.7% inhibition of angiotensin converting enzyme 2 (ACE2) at 25 μg/mL [67]. Isolated from a deep-sea floor, the *Streptomyces* sp. GGS53 produced two pyrrolosesquiterpenes glaciapyrroles, D and E (**107** and **108**, Figure 7) [68]. Influenza A viruses were used to infect Mardin–Darby canine kidney cells; **108** exhibited significant antiviral activity, resulting in the reduction of the viral titer by 70%.

## 4. Oxazoles and Thiazoles

**Figure 11:**
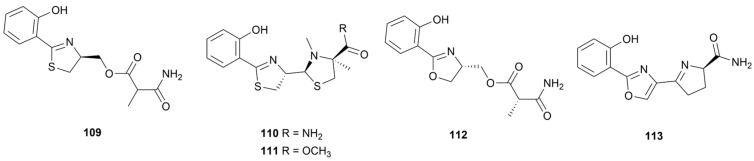
Chemical structures of oxazole and thiazole alkaloids **109**–**113** isolated from marine *Streptomyces*.

Oxazole and thiazole rings exhibit a wide range of pharmacological activities, such as antiproliferative, anti-inflammatory and antimicrobial activity, by binding non-covalently to many enzyme and receptor targets [69,70]. Most of these alkaloids have been mentioned in other chapters, and this chapter summarizes the remaining five compounds **109**–**113** (Figure 11). Under LC-MS/MS molecular networking guidance, five siderophores containing oxazole or thiazole rings were isolated from *S. diastaticus* NBU2966 collected from marine sponge *Axinellida* sp. [71]. However, none of these metabolites demonstrated activity against *S. aureus*, MRSA, *B. subtilis*, and *P. aeruginosa*.

## 5. Pyridines

Pyridine alkaloids are a class of important skeletons for maintaining the pharmacological activity of drugs [72]. This chapter summarizes pyridine and its derivatives of marine *Streptomyces* sp., including pyridone, benzopyridine (quinoline), and the saturated variant, piperidine.

### 5.1. Piericidins

**Figure 12:**
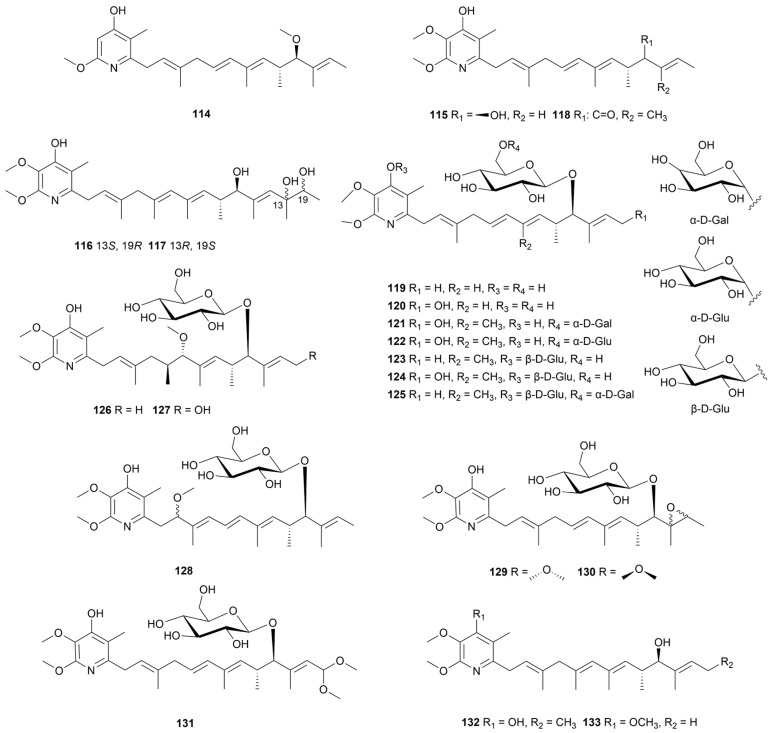
Chemical structures of piericidin analogues **114**–**133** isolated from marine *Streptomyces*.

Piericidins are a class of 4-pyridinol alkaloids decorated with methylated polyene side chains, mostly isolated from actinomycetes of soil, marine or bio-symbiotic origin [73]. Due to structural similarities with coenzyme Q, some piericidins exhibit NADH−ubiquinone oxidoreductase inhibitory activity [74]. Moreover, insecticidal, cytotoxic, and bactericidal effects of piericidins have been reported. When a mangrove soil-derived *Streptomyces* sp. CHQ-64 was knocked out of the gene *rdmF*, a positive regulatory gene for reedsmycins (polyol polyene macrolides), one mutant strain was shown to produce a piericidin analogue (**114**, Figure 12), which displayed prominent cytotoxicity against the HeLa, NB4, A549 and H1975 cell lines (IC_50_ values of 0.003–0.56 μM). Seventeen piericidin derivatives (**115**–**131**, Figure 12) purified from *S. psammoticus* SCSIO NS126 from mangrove sediment samples showed strong or moderate activity against renal cell (RCC) carcinoma cell line ACHN with IC_50_ values of 0.31–60 μM [75]. A mechanism-of-action (MOA) study revealed that compounds **115**–**131** could increase the expression of peroxiredoxin 1 (PRDX1), decreasing the level of reactive oxygen species in cells. The piericidin glycosides (**119**–**131**) were more effective in binding to PRDX1 than the aglycones (**115**–**118**), although ultimately there was no significant difference in antitumor activity. Piericidin A5 (**132**) together with G1 (**133**) produced by strain SCSIO 40063 exhibited antitumor activity against SF-268, MCF-7, HepG2 and A549 tumor cell lines, with IC_50_ values ranging from 10.0 to 12.7 μM [76].

### 5.2. Quinolines

**Figure 13:**
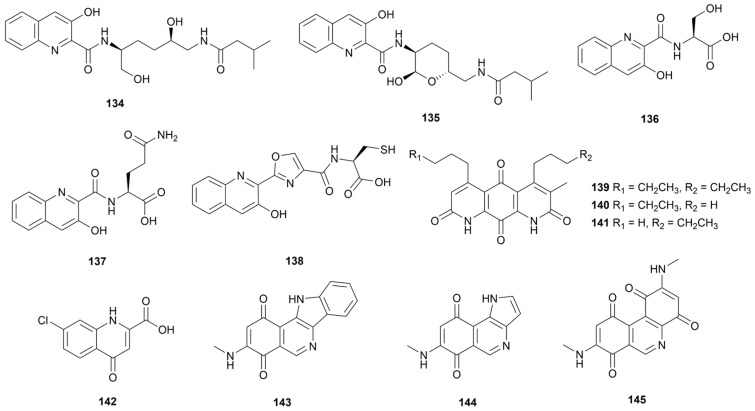
Chemical structures of quinolines **134**–**145** isolated from marine *Streptomyces*.

Quinolines have been designed as important skeletons in drug structures for more than two centuries [77]. Classic examples are quinine, the first effective antimalarial drug in history, and the broad-spectrum antibiotic fluoroquinolone [78]. This subsection provides an overview of quinoline alkaloids of marine *Streptomyces* sp. origin and their derivatives isoquinolines and quinolones. Compounds **134**–**141** (Figure 13) were identified as simple quinoline alkaloids purified from marine *Streptomyces*. Strain CNP975 produced two rare quinoline derivatives containing 3-hydroxyquinaldic acid (3HQA) fragments, named actinoquinolines A and B (**134** and **135**), with stronger inhibitory activity against cyclooxygenases-2 (COX-2, IC_50_ of 2.13 and 1.42 μM, respectively) compared to cyclooxygenases-1 (COX-1, IC_50_ of 7.6 and 4.9 μM, respectively) [79]. The cyclization of the hydroxyl group increases the activity of the compounds against COX-1/2. Three amino-acid-substituted quinoline derivatives (**136**–**138**) were isolated by *S. cyaneofuscatus* M-157 collected from coral samples containing serine, glutamine, and cysteine residues unit, respectively [80]. Only compound **137** displayed weak cytotoxicity against human tumor cell line HepG2, with IC_50_ value of 51.5 µM. Diazaquinomycins E–G (**139**–**141**) were unusual diazaanthracene alkaloids, and compound **139,** with double substitution, had cytotoxic activity against the ovarian cancer cell line OVCAR5 by upregulating the cell cycle inhibitor p21 and impairing DNA (IC_50_ value of 9.0 μM) [81]. Antichlamydial activity-guided purification of a chlorinated quinolone ageloline A (**142**, Figure 13) was isolated from *Streptomyces* sp. SBT345 collected from the Mediterranean sponge *Agelas oroides* [82]. Compound **142** dose-dependently exhibited inhibition of *Chlamydia trachomatis* growth (IC_50_ value of 9.54 ± 0.36 μM) by inhibiting reactive oxygen species (ROS) production during the early stages of infection. The high-yield extract medium of strain B1848 afforded three isoquinolinequinone alkaloids, mansouramycins E–G (**143**–**145**, Figure 13) [83]. In a cytotoxicity assay against 36 tumor cells, compound **144** exhibited selective moderate cytotoxic activity (mean IC_50_ value of 7.92 µM), while compound **143** exhibited weak effect.

### 5.3. Other Pyridines

**Figure 14:**
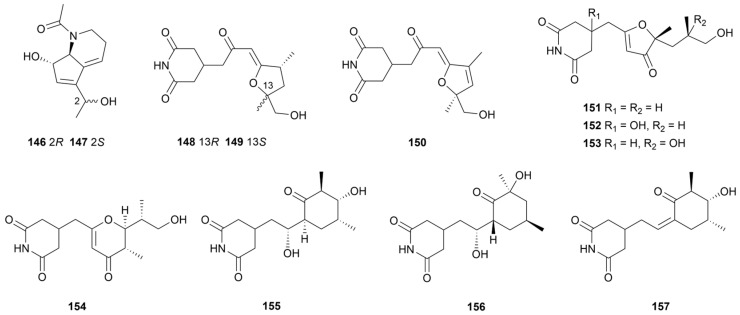
Chemical structures of other pyridines **146**–**157** isolated from marine *Streptomyces*.

Two pyridine derivatives, strepchazolins A and B (**146** and **147**, Figure 14), were purified from *S. chartreusis* NA02069 [84]. Compound **146** inhibited a 64.0 µM MIC value against *B. subtilis* and a 50.6 µM IC_50_ value against acetylcholinesterase. Compound **147** was inactive, indicating that steric configuration affected biological activity. Isolated from a marine mud sample, *Streptomyces* sp. ZZ741 afforded ten glutarimide analogues named streptoglutarimides A−J (**148**–**157**, Figure 14) [85]. All analogues were effective against MRSA (MIC = 9–11 μg/mL), *E. coli* (MIC = 8–12 μg/mL) and *C. albicans* (MIC = 8–20 μg/mL). The five-membered and six-membered rings in C-4 substituents had no significant effect on pharmacological activity. Moreover, **155** displayed promising antiproliferative activity against glioma cells U87MG and U251 with IC_50_ values of 3.8 ± 0.6 µM and 1.5 ± 0.1 µM, respectively.

## 6. Pyrazines and Piperazines

### 6.1. Pyrazines

**Figure 15:**
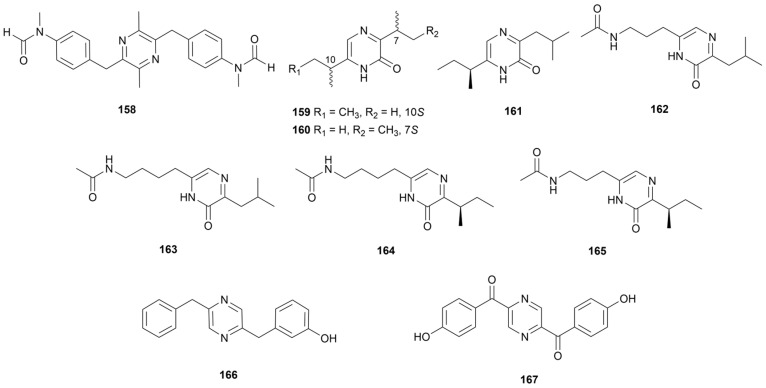
Chemical structures of pyrazines **158**–**167** isolated from marine *Streptomyces*.

Owing to nitrogen atoms acting as hydrogen bonding acceptors and the structure being conducive to nucleophilic reaction, pyrazine is commonly used as a classical pharmacophore [86]. Pyrazine derivatives have been reported for applications as antitumor drugs, diuretics, anti-inflammatory and anti-infective drugs [87]. Griseusrazin A (**158**, Figure 15) was isolated from a strain *S. griseus* subsp. *griseus* 09-0144 and activated the expression of heme oxygenase 1 which inhibits the upstream NF-κB pathway [88]. Therefore, it could downregulate the expression of related enzymes inducible nitric oxide synthase (iNOS) and COX-2 at the transcriptional level as well as the production of inflammatory mediators NO and PGE_2_. Compounds **159**–**161** (Figure 15) were purified by *Streptomyces* sp. Did-27 isolated from the marine tunicate *Didemnum* sp., from which **159** and **161** showed weak cytotoxicity against HCT-116 and MCF-7 cancer cell lines with IC_50_ of 25–35 µg/mL [89]. Collected from a sample of coastal soil from Zhoushan Islands, China, the *Streptomyces* sp. ZZ446 afforded four pyrazinones of streptopyrazinones A–D (**162**–**165**, Figure 15) [90]. These compounds exhibit 35.0-60.0 µg/mL MIC values against *C. albicans* and 58.0-65.0 µg/mL MIC values against MRSA. In 2022, two pyrazines named actinopolymorphols E and F (**166** and **167**, Figure 15) were obtained from marine sediment-derived strain CNP-944 and only compound **167** exhibited weak activity against *K. rhizophila*, *B. subtilis* and *S. aureus* with MIC values of 16–64 µg/mL [91]. Compound **166** was inactive, which indicated that the carbonyl was the key active moiety.

### 6.2. Diketopiperazines

Piperazine alkaloids of biological origin are most commonly of the 2,5-diketopiperazines (2,5-DKPs) type, with a cyclodipeptide structure formed by condensation of two amino acids [92]. These simple dipeptides reported for multiple biological activities have a flexible skeleton with multiple chiral centers and four hydrogen bonding sites [93].

**Figure 16:**
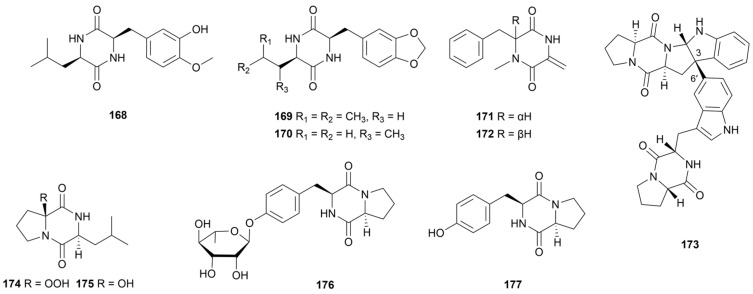
Chemical structures of diketopiperazines **168**–**177** isolated from marine *Streptomyces*.

A variety of 2,5-DKPs alkaloids (**168**–**177**, Figure 16) had been isolated and characterized from several marine *Streptomyces* spp. Compounds **168**–**170** were condensed with leucine and phenylalanine residues and purified by a *Streptomyces* sp. MNU FJ-36 obtained from the intestinal fabric of *Katsuwonus* sp. [94]. All compounds were weakly inhibitory to A549 and HCT-116 cell lines. Streptodiketopiperazines A (**171**) and B (**172**) containing phenylalanine residues were isolated from the Mariana Trench source *Streptomyces* sp. SY1965 [95]. Biological evaluation showed that **171** and **172** showed weak antifungal activity against *C. albicans* (MIC = both 42 μg/mL). A 2,5-DKPs dimer naseseazine C (**173**) had moderate inhibitory activity against chloroquine-sensitive *Plasmodium falciparum* (average IC_50_ value = 3.52 ± 1.2 µM) [96]. This dimer was connected by an unconventional C-6′/C-3 linkage and thus promoted antimalarial activity. Actinozine A (**174**) and cyclo(2-OH-D-Pro-L-Leu) (**175**) as two 2,5-DKP alkaloids were produced by *Streptomyces* sp. Call-36 from the Red Sea sponge *Callyspongia* sp. [97]. Compound **174** had a special hydroperoxy moiety on the proline residue. Antimicrobial assays against *S. aureus* and *C. albicans* revealed that **174** and **175** showed inhibition zones of 16-23 mm. Furthermore, a glycosylated 2,5-DKP (**176**) and its aglycone (**177**) showed inhibitory activity against MRSA, *E. coli* and *C. albicans* (MIC = 26.0–37.0 µg/mL) [98].

## 7. Phenazines and Phenoxazines

**Figure 17:**
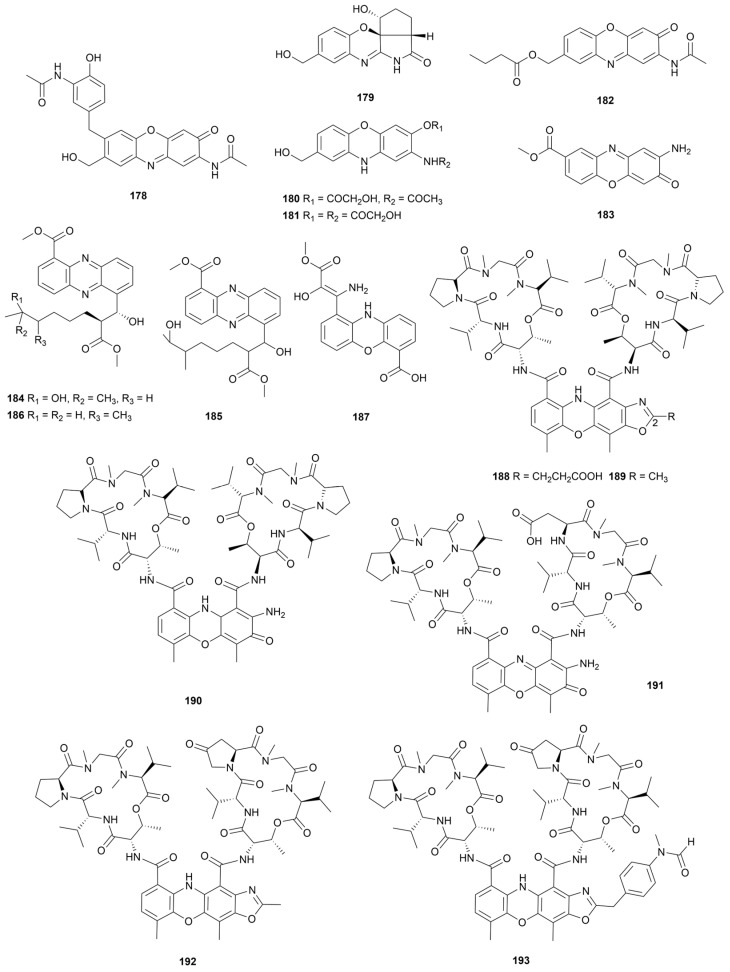
Chemical structures of phenazine and phenoxazine alkaloids **178**–**193** isolated from marine *Streptomyces*.

To the best of our knowledge, phenazines and phenoxazines are mainly derived from SMs of *Streptomyces* and *Pseudomonas* isolated from soil or marine habitats [99]. Most of these alkaloids are characterized by promising biological activities such as antibacterial, antiviral, antitumor and antiparasitic effects [100]. Six antitumor phenoxazines venezuelines A–E (**178**–**182**, Figure 17) and maroxazinone (**183**, Figure 17) were isolated from two sediments-derived *Streptomyces* [101,102]. Compound **179** showed moderate antitumor activity against five cancer cell lines with IC_50_ values of 5.74–9.67 μM and weak activity against human hepatoma cell Bel 7042 (IC_50_ >10 μM). Notably, the cytotoxicity of this compound may be explained by significant upregulation of the orphan nuclear receptor Nur77 (apoptosis-associated) expression. **183** showed moderate antiproliferative activity against MCF7, HEPG2 and HCT116 cell lines with IC_50_ values of 4.32, 2.90 and 8.51 μg/mL, respectively. Cytotoxic activity of phenoxazinones was stronger than phenoxazines and the increase of substituents weakened the activity. Sponges can host microorganisms colonization due to their porous structure, therefore the metabolites of sponge symbiotic microorganisms are important sources of marine natural products [103]. *Streptomyces* sp. HB202 was isolated from the sponge *Halichondria panicea* and yielded three phenazine alkaloids streptophenazines I–K (**184**–**186**, Figure 17) [104]. These compounds inhibited the activity of inflammatory response associated enzyme phosphodiesterase (PDE 4B) with IC_50_ values ranging from 11.6 to 12.2 μM. In addition, compound **186** had antibacterial activity against *B. subtilis* and *S. epidermidis* (IC_50_ = 21.6 ± 6.8 µM and 14.5 ± 2.0 µM, respectively).

One phenoxazine derivative strepoxazine A (**187**, Figure 17) was produced by a strain SBT345 obtained from the mediterranean sponge *Agelas oroides* [105]. The IC_50_ value for **187** against promyelocytic leukemia cells HL-60 was 16 µg/mL. Actinomycin analogues (**188**–**193**, Figure 17) are a class of tetracyclic 5H-oxazolo[4,5-b]phenoxazine alkaloids [106,107]. Neo-actinomycin A (**188**) exhibited promising cytotoxic activity against HCT116 and A549 cancer cell lines (IC_50_ = 38.7 nM and 65.8 nM, respectively), as well as the biosynthetic pathways of **188** and **189** were the condensation of actinomycin D (**190**) with α-ketoglutarate or pyruvate. Actimomycin S (**191**) and neo-actinomycins C–D (**192**–**193**) were bacteriostatic against five common pathogenic bacteria (MIC = 2.5–80.0 μg/mL) and exhibited potent cytotoxic activity against HepG2 liver carcinoma cell line by blocking the G0/G1 phase cell cycle. The different substituents at C-2 position of oxazole ring showed great difference in biological activity.

## 8. Indolizidines and Pyrrolizidines

### 8.1. Indolizidines

Indolizidine were reported to have broad biological activity, such as antitumor activity, anti-infective system disease activity, and anti-inflammatory activity [108]. Most indolizidines are obtained from plants and animals, rarely from microbial sources [109].

**Figure 18:**
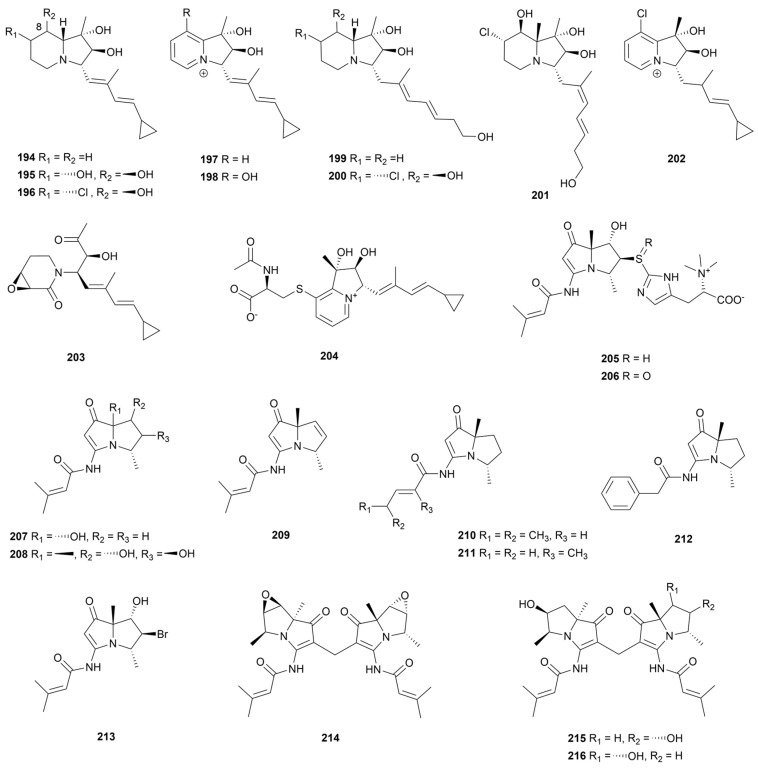
Chemical structures of indolizidine and pyrrolizidine alkaloids **194**–**216** isolated from marine *Streptomyces*.

Eight indolizidine alkaloids cyclizidines B–I (**194**–**201**, Figure 18) were detected in the EtOAc extracts of the strain *Streptomyces* sp. HNA39 [110]. Cyclizidine C (**195**) showed the most promising activity against PC-3 and HCT-116 cancer cell lines, with IC_50_ values of 0.52 ± 0.03 μM and 8.3 ± 0.1 μM, respectively. Moreover, compounds **195**, **198**, **200** and **201** exhibited moderate inhibitory activities against protein kinase ROCK2. In another report, a low-yielding indolizidine named cyclizidine J (**202**, Figure 18) was detected in strain HNA39 [111]. This compound had an uncommon chlorine atom substitution at the C-8 position. However, **202** lacked inhibitory activity against cancer cell line PC-3 and protein kinase. A stress culture of marine hydrothermal vent actinomycetes with heavy metal ions can activate silent biosynthetic pathways [112]. After the addition of 100 μmol/L Ni^2+^ to the medium of metal-resistant *Streptomyces* sp. WU20, a cyclizidine analogue (**203**, Figure 18) that was absent before addition was purified [113]. The authors hypothesized that the ring opening of the five-membered ring in the structure of alkaloid **203** was due to the inhibition of normal biosynthesis by heavy metal stress. Compound **203** was bacteriostatic against *B. subtilis* with MIC of around 32 μg/mL. Chemical analysis of symbiotic strain *Streptomyces* sp. HZP-2216E from fresh sea lettuce *Ulva pertusa* led to the discovery of an indolizinium alkaloid, streptopertusacin A (**204**, Figure 18), which had a 40 mg/mL MIC value against MRSA [114]. The antitumor activity of alkaloids was enhanced when C-8 was replaced by a hydroxyl group, but the cytotoxicity was lost after the aromatization of the indolizidine core.

### 8.2. Pyrrolizidines

Pyrrolizidines are mainly derived from plants as toxic components of chemical defense [115]. Bacterial-derived pyrrolizidines have been reported less frequently, with a total of 12 species and about 60 compounds of this class identified as of 2021 [116]. These Pyrrolizidines are commonly biosynthesized by multidomain NRPS gene clusters and are post-modified as well by flavine adenosine dinucleotide (FAD)-dependent monooxygenases [117].

Bohemamine is a rare pyrrolizidine subtype derived only from actinomycetes [118]. The fermentation broth of strain *S. spinoverrucosus* SNB-048 purified two bohemamine-type pyrrolizidines named spithioneines A and B (**205** and **206**, Figure 18) with rare ergothioneine moiety [119]. In the same year, six derivatives of bohemamines D−I (**207**–**212**, Figure 18) were again isolated from strain SNB-048 [120]. Unfortunately, none of the compounds showed significant activity. Tracing the cytotoxic activity of *S. spinoverrucosus* SNB-032 metabolites led to the isolation of an analogue, 5-Br-bohemamine C (**213**, Figure 18), as well as three dimeric bohemamines dibohemamines A–C (**214**–**216**, Figure 18) [121]. The authors confirmed that the dimer formation was a non-enzymatic Baylis–Hillman addition reaction of monomeric compounds using formaldehyde in the medium. Compounds **215** and **216** exhibited potent cytotoxicity against an NSCLC cell line A549 with IC_50_ values of 0.140 and 0.145 µM, respectively. In addition, compound **216** showed moderate activity against an HCC1171 cell line (IC_50_ = 1.2 µM). Bohemamine-type pyrrolizidines exhibited significant cytotoxic activity due to the polymerization of dimer.

## 9. Amides

### 9.1. Linear Amides

**Figure 19:**
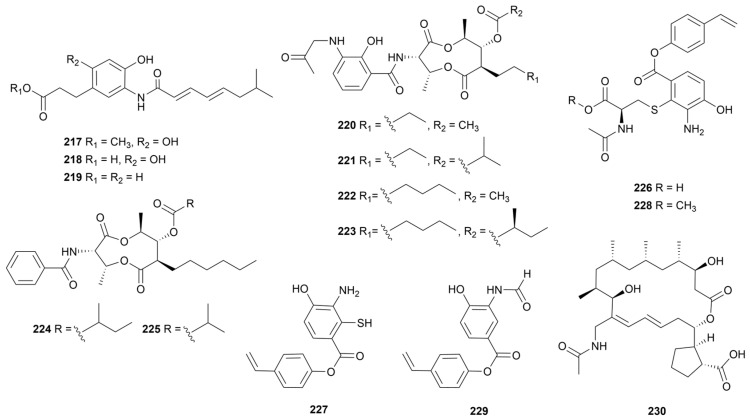
Chemical structures of linear amides **217**–**230** isolated from marine *Streptomyces*.

Marine sediment-derived *Streptomyces* sp. SNE-011 afforded three acylated arylamine alkaloids named carpatamides A–C (**217**–**219**, Figure 19) [122]. Compounds **217** and **219** displayed positive activity against HCC366, A549 and HCC44 cell lines (IC_50_ = 2.2–8.4 μM). For compound **218**, the authors hypothesized that the reason for its inactivity was the inability of the structure to pass through the cell membrane. Antimycin is an antibiotic with antibacterial, insecticidal and anticancer activity, consisting of a rare nine-membered dilactone core [123]. Antimycins E–H (**220**–**223**, Figure 19) were isolated from *Streptomyces* sp. THS-55, and showed extremely significant cytotoxic activity against the HeLa cell line (IC_50_ < 0.1 μM) by downregulating the levels of E6/E7 oncoproteins [124]. The potency was dependent on the long-chain substituent of R_2_ and the acyl group of R_3_. In addition, neoantimycins A and B (**224** and **225**, Figure 19) were isolated from *S. antibioticus* and exhibited weak cytotoxic activity against the SF-268 cancer cell line [125]. Bagremycin is a phenol ester formed from *p*-hydroxystyrene and *p*-hydroxybenzoic acid with antimicrobial activity [126]. Bagremycins C and D (**226** and **227**, Figure 19) were isolated in 2017 from *Streptomyces* sp. Q22 [127]. The following year, bagremycins F and G (**228** and **229**, Figure 19) were purified from coastal mud-sourced *Streptomyces* sp. ZZ745 [128]. Compound **226** inhibited the G_0_/G_1_ cell cycle in four glioma cells (U87MG, U251, SHG44 and C6) with IC_50_ values of 2.2 to 6.4 μM. Furthermore, compounds **228** and **229** showed 41.8 and 67.1 μM MIC values against *E. coli*. One N-acetyl macrolide analogue N-acetylborrelidin B (**230**, Figure 19) was detected by a strain *S. mutabilis* MII with stronger activity against *Staphylococcus warneri* (18 mm zone of inhibition) [129].

### 9.2. Macrolactams

Macrolactams are a class of macrocyclic compounds in which amide units are integrated into a polyketide skeleton above twelve carbons [130]. These compounds often contain an azacyclic core skeleton or azacyclic substituent modifications that result in alkalinity [131].

**Figure 20:**
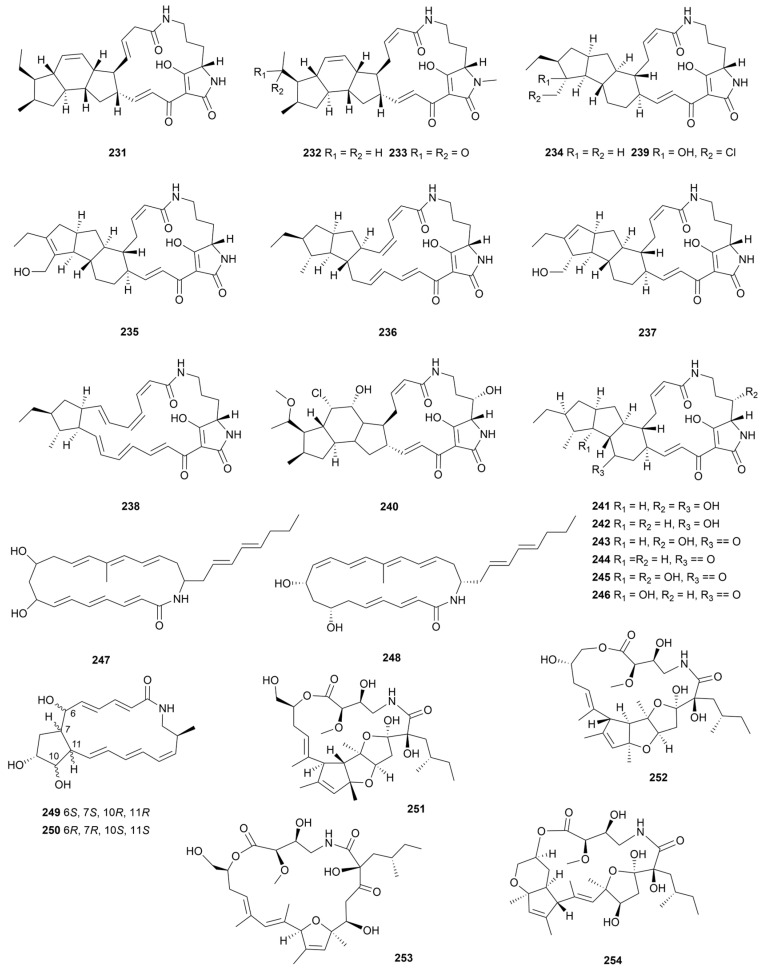
Chemical structures of macrolactams **231**–**254** isolated from marine *Streptomyces*.

Polycyclic tetramate macrolactams (PTMs) are polycyclic macrolactam examples encoded by the PKS/NRPS heterozygous gene cluster [132]. Three PTMs (**231**–**233**, Figure 20) were purified from *S. zhaozhouensis* CA-185989 [133]. Compounds **231** and **232** had the most promising activity against MRSA, *C. albicans* and *A. fumigatus* (MIC = 1–8 μg/mL). The addition of the strong promoter ermE*p to the PTM gene cluster of the deep-sea-derived *S. pactum* SCSIO 02999 activated the generation of six antitumor active PTMs, pactamides A–F (**234**–**239**, Figure 20) [134]. Compound **234** exhibited 0.24–0.51 μM IC_50_ values against four cancer cell lines. In addition, some atypical structural PTMs (Figure 20) with moderate cytotoxic activity, such as chlorinated derivatives chlokamycin (**240**) and H-10/H-11 *trans*-oriented PTM (**241**–**246**), have been reported in recent years from marine *Streptomyces* metabolites [135,136]. The substituent class of the side-chain tricyclic ring significantly affected the biological activity of the compounds. Moreover, a simple macrolactam JBIR-150 (**247**, Figure 20) exhibited cytotoxic activity against human malignant mesothelioma MESO-1 (IC_50_ = 2.3 μM) and human T-lymphoma Jurkat cells (IC_50_ = 0.9 μM) [137]. Another similar substance, muanlactam (**248**, Figure 20) was targeted for purification from *Streptomyces* sp. MA159 through combined genomic library and spectral characterization; and its IC_50_ value against the HCT116 cell line was 1.58 μM [138]. *Streptomyces* sp. OUCMDZ-4348 is an extreme habitat microbe collected from Antarctica and was shown to produce two bicyclic macrolactams (**249** and **250**, Figure 20) [139]. Only **249** exhibited a moderate IC_50_ value (9.8 µM) against the gastric carcinoma cell line N87. In another polar actinomyces, *S. somaliensis* 1107, four macrocyclic lactam containing furan rings (**251**–**254**, Figure 20) were isolated, one of which, compound **251** had anti-inflammatory activity [140].

## 10. Miscellaneous Alkaloids

**Figure 21:**
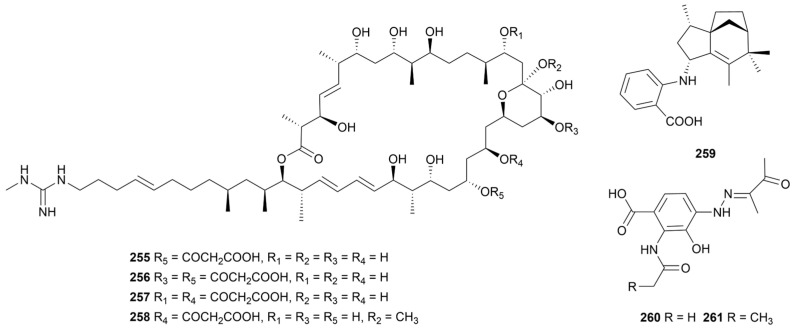
Chemical structures of miscellaneous alkaloids **255**–**261** isolated from marine *Streptomyces*.

Niphimycin (NM) is a class of guanidylpolyol macrolide antibiotics with extensive antibacterial activity against fungi and Gram-positive bacteria [141]. Four niphimycins derivatives (**255**–**258**, Figure 21) were obtained from *Streptomyces* sp. IMB7-145 and **255**, **257** and **258** displayed antibacterial effects on MRSA and vancomycin-resistant enterococci (VRE) with MIC values of 8–64 μg/mL [142]. Moreover, compound **255** exhibited significant anti-*M. tuberculosis* activity (MIC = 32 μg/mL) and significantly inhibited the growth of the phytopathogenic fungus *Fusarium oxysporum* f. sp.*cubense* (EC_50_ = 1.20 μg/mL) as well as demonstrating cytotoxic activity against nasopharyngeal carcinoma cell lines TW03 and 5-8F (IC_50_ = 12.24 µg/mL and 9.44 µg/mL, respectively) [143,144]. The increase in malonyl substituents had a negative effect on its biological activity. Antartin (**259**, Figure 21) was a zizaane-type sesquiterpene produced by strain *Streptomyces* sp. SCO736 and showed promising cytotoxic activity against twelve human cancer cells, with 50% growth inhibition (GI_50_) of 4–8 µg/mL and inhibited the production of solid lung tumor cells [145]. Penzonemycins A and B (**260**–**261**, Figure 21) were two phenylhydrazones, in which their hydrazone moiety was synthesized by a non-enzymatic Japp−Klingemann coupling reaction [146]. Compound **260** inhibited cancer cell lines SF-268, MCF-7, A549 and HepG-2, with IC_50_ values ranging from 30.44 to 61.92 µM.

## 11. Conclusions and Future Perspectives

Microorganisms in special habitats have cryptic and extraordinary potential for biosynthesizing unique SMs with diverse biological properties. Marine *Streptomyces* as an excellent producer of therapeutic agents has become a global hotspot in natural product research and continue to play a paramount role in the production of new alkaloids for drug discovery. This review comprehensively summarizes as many as 261 new alkaloids discovered in marine *Streptomyces* in the past decade (January 2013–June 2023). Among these metabolites, 199 compounds have promising therapeutic effects. For instance, compounds **38**, **41**, **42**, **97**, **98**, **142** and **193** exhibited excellent antimicrobial activity, and **114**, **188** and **220**–**239** displayed remarkable cytotoxic effects. In addition, compounds **5**–**16** showed significant selective inhibition on protein kinases PKC, ROCK2 and BTK. This work will pave the way for further development of marine *Streptomyces*-derived alkaloids.

Although strategies for the isolation of marine microbial metabolites have been revolutionized over the past decades, there are still difficulties to overcome. Traditional means of separating natural products are subject to randomization. The efficient isolation of novel natural products and removal of inactive known compounds have been thorny issues hindering the development of natural medicinal chemistry. The annotation of BGCs, as well as LC-MS/MS-based metabolite structure prediction methods, will remain hot research topics in this field in the future. BGCs of marine microorganisms often have low or no expression under routine laboratory culture conditions. BGC activation techniques, such as OSMAC strategy, strain co-culture, ribosome engineering, heterologous expression of gene clusters and overexpression/knockout of regulatory genes and ribosome engineering, provide methodological references to break this bottleneck. In addition to the discovery of these therapeutic agents, it is crucial to solve the problem of compound supply. Currently, in addition to the total synthesis route design of natural products, the modification of industrial production strains using genetic engineering approaches and optimization of microbial fermentation and extraction, as well as purification processes at various levels, should be employed in the preparation of these substances.

## Data Availability

The data presented in this study are available in the Supplementary Material.

## Supplementary Materials

The following supporting information can be downloaded at: https://www.mdpi.com/article/10.3390/md22010051/s1, Table S1: detailed information for new alkaloids from marine *Streptomyces* discovered in 2013–June 2023.
