# Supplementary material for: Marine Streptomyces-Derived Novel Alkaloids Discovered in the Past Decade
Source: Mar Drugs. 2024 Jan 22;22(1):51. doi: 10.3390/md22010051 (PMC10821133; doi:10.3390/md22010051)
Supplement: Supplementary file 1 [file marinedrugs-22-00051-s001.zip › marinedrugs-2804693-supplementary.pdf]

Table S1: Detailed information for new alkaloids from marine *Streptomyces* discovered in 2013-June 2023

No.	Compound name	Type	Strain No.	Source	Bioactivity	Ref.
**1**	12-*N*-methyl-k252c	Staurosporine (Bisindole)	*Streptomyces* sp. A22	Marine sediments of Ningbo City (Zhejiang province, China, N 29°28′ E 121°57′)	Enzyme inhibition against three protein kinases PKC, BTK and ROCK2 with IC_{50} values of 1.84, 1.51 and 0.96 μM, respectively.	[1]
**2**	9-hydroxy-K252c	Staurosporine (Bisindole)	*Streptomyces* sp. DT-A61	A sediment sample of Dongtou, Zhejiang Province, China (N 27°87′01.67″ E 121°16′99.45″)	Cytotoxic activity against PC-3 cell line with an IC_{50} value of 8.0 μM. Enzyme inhibition against three protein kinases PKC, ROCK2 and ASK1 with IC_{50} values of 0.98, 0.34 and 0.74 μM, respectively.	[2]
**3**	3-hydroxy-K252c				Cytotoxic activity against PC-3 cell line with an IC_{50} value of 21 μM. Enzyme inhibition against three protein kinases PKC, ROCK2 and ASK1 with IC_{50} values of 3.2, 0.0057 and 3.0 μM, respectively.	
**4**	3-hydroxy-7-methoxy-K252c				Cytotoxic activity against PC-3 cell line with an IC_{50} value of 18 μM. Enzyme inhibition against three protein kinases PKC and ROCK2 with IC_{50} values of 1.4 and 0.91 μM, respectively.	
**5**	3′-epi-*N*-acetyl-holyrine A	Staurosporine (Bisindole)	*Streptomyces* sp. A68	Sediments of Dongtou County (N27°49′04.44″E121°07′55.33″, Zhejiang province, China)	Cytotoxic activity against human prostate cancer PC3 cell lines with an IC_{50} value of 0.8 μM Enzyme inhibition against three protein kinases PKC, ROCK2 and BTK with IC_{50} values of 0.17, 0.21 and 1.45 μM, respectively.	[3]
**6**	3′-*N*-acetyl-holyrine A				Cytotoxic activity against human prostate cancer PC3 cell lines with an IC_{50} value of 16.2 μM. Enzyme inhibition against three protein kinases PKC, ROCK2 and BTK with IC_{50} values of 0.91, 2.70 and 0.35 μM, respectively.	
**7**	3′-*N*-formyl-holyrine A				Cytotoxic activity against human prostate cancer PC3 cell lines with an IC_{50} value of 1.5 μM.	

					Enzyme inhibition against three protein kinases PKC, ROCK2 and BTK with IC_{50} values of 1.04, 2.15 and 1.24 μM, respectively.	
**8**	7-oxo-holyrin A	Staurosporine (Bisindole)	*Streptomyces* sp. NB-A13	Marine sediments of Ningbo City (Zhejiang province, China, N 29°28.4239′E121°57.6236′)	Cytotoxic activity against PC-3 and SW-620 cell lines with IC_{50} values of 4.03 ± 0.29 and 2.14 ± 0.08 μM, respectively.<br>Enzyme inhibition against PKC-θ protein kinases with an IC_{50} value of 1.35 μM.	[4]
**9**	4′-*N*-formyl-7-oxo-holyrin A				Cytotoxic activity against PC-3 and SW-620 cell lines with IC_{50} values of 2.05 ± 0.06 and 0.74 ± 0.01 μM, respectively.<br>Enzyme inhibition against PKC-θ protein kinases with an IC_{50} value of 4.48 μM.	
**10**	3′-(hydroxyl(oxiran-2-yl)methoxy)-holyrine A				Cytotoxic activity against PC-3 and SW-620 cell lines with IC_{50} values of 2.45 ± 0.08 and 2.00 ± 0.19 μM, respectively.<br>Enzyme inhibition against PKC-θ protein kinases with an IC_{50} value of 5.75 μM.	
**11**	3′-epi-5′-methoxy-K252d				Cytotoxic activity against PC-3 and SW-620 cell lines with IC_{50} values of 16.60 ± 0.43 and 9.54 ± 0.65 μM, respectively.<br>Enzyme inhibition against PKC-θ protein kinases with an IC_{50} value of 9.435 μM.	
**12**	3′-*epi*-K252d	Staurosporine (Bisindole)	*Streptomyces* sp. DT-A65	Marine sediments of Dongtou Island (Zhejiang province, China, N 27°500′ E 121°100′)	Cytotoxic activity against PC-3 cell line with an IC_{50} value of 9.67 ± 0.37 μM.<br>Enzyme inhibition against protein kinases PKC and BTK with IC_{50} values of 0.25 ± 0.03 and 1.91 ± 0.07 μM, respectively.	[5]
**13**	2′,4′-*epi*-K252d				Cytotoxic activity against PC-3 cell line with an IC_{50} value of 9.67 ± 0.37 μM.<br>Enzyme inhibition against protein kinases PKC and BTK with IC_{50} values of 0.35 ± 0.02 and 0.39 ± 0.03 μM, respectively.	

**14**	9-hydroxy-3′-N-acetylholyrine A	Staurosporine (Bisindole)	*Streptomyces* sp. DT-A61	A sediment sample of Dongtou, Zhejiang Province, China (N 27°87′01.67″ E 121°16′99.45″)	Enzyme inhibition against three protein kinases PKC and ROCK2 with IC_{50} values of 0.097 and 1.4 μM, respectively.	[2]
**15**	3-hydroxy-3′-N-acetylholyrine A				Cytotoxic activity against PC-3 cell line with an IC_{50} value of 3.6 μM. Enzyme inhibition against three protein kinases PKC, ROCK2 and ASK1 with IC_{50} values of 0.46, 0.04 and 6.4 μM, respectively.	
**16**	3-hydroxyholyrine A				Cytotoxic activity against PC-3 cell line with an IC_{50} value of 3.1 μM. Enzyme inhibition against three protein kinases PKC, ROCK2 and ASK1 with IC_{50} values of 0.079, 0.15 and 1.1 μM, respectively.	
**17**	3-hydroxy-K252d	Staurosporine (Bisindole)	*Streptomyces* sp. OUCMDZ-3118	A piece of deep-sea sediment (2061 m) collected in the South China Sea	Cytotoxic activity against A549 and MCF-7 cell lines with IC_{50} values of 1.2 ± 0.05 μM and 1.6 ± 0.09 μM, respectively.	[6]
**18**	Streptocarbazoles C	Staurosporine (Bisindole)	*Streptomyces* sp. DT-A65	Marine sediments of Dongtou Island (Zhejiang province, China, N 27°500′ E 121°100′)	Cytotoxic activity against PC-3 cell line with an IC_{50} value of 41.3 ± 2.38 μM. Enzyme inhibition against protein kinases BTK with an IC_{50} value of 1.43 ± 0.13 μM.	[5]
**19**	Streptocarbazole D	Staurosporine (Bisindole)	*Streptomyces* sp. DT-A61	A sediment sample of Dongtou, Zhejiang Province, China (N 27°87′01.67″ E 121°16′99.45″)	Enzyme inhibition against three protein kinases PKC, ROCK2 and ASK1 with IC_{50} values of 2.1, 0.97 and 4.1 μM, respectively.	[2]
**20**	Streptocarbazole E				Cytotoxic activity against PC-3 cell line with an IC_{50} value of 5.6 μM. Enzyme inhibition against three protein kinases PKC and ROCK2 with IC_{50} values of 1.4 and 0.34 μM, respectively.	
**21**	Streptocarbazole F	Staurosporine (Bisindole)	*Streptomyces* sp. OUCMDZ-5380	A driftwood sample collected at the Beibu Gulf nearby Guangxi, China (N 21°44′39″ E 108°35′22″)	Cytotoxic activity against MV4-11 cancer cell with an IC_{50} value of 0.81 ± 0.007 μM.	[7]
**22**	Streptocarbazole G				Cytotoxic activity against MV4-11 cancer cell with an IC_{50} value of 0.55 ± 0.04 μM.	

**23**	Streptocarbazole H				Cytotoxic activity against MV4-11 cancer cell with an IC_{50} value of 1.88 ± 0.11 μM.	
**24**	3′-O-demethyl-4′-N-demethyl-4′-N-acetyl-4′-epi-staurosporine	Staurosporine (Bisindole)	*Streptomyces* sp. DT-A61	A sediment sample of Dongtou, Zhejiang Province, China (N 27°87′01.67″ E 121°16′99.45″)	Cytotoxic activity against PC-3 cell line with an IC_{50} value of 0.16 μM. Enzyme inhibition against three protein kinases PKC, ROCK2 and ASK1 with IC_{50} values of 0.092, 0.26 and 0.77 μM, respectively.	[2]
**25**	Indimicin A	Halogenated bisindole	*Streptomyces* sp. SCSIO 03032	deep-sea	-	[8]
**26**	Indimicin B				Cytotoxic activity against MCF-7 cell line with an IC_{50} value of 10.0 μM.	
**27**	Indimicin C				-	
**28**	Indimicin D				-	
**29**	Indimicin E				-	
**30**	Lynamicin F				-	
**31**	Lynamicin G				-	
**32**	Spiroindimicin G	Halogenated bisindole	*Streptomyces* sp. SCSIO 03032 Δ*spmH*	deep-sea	Cytotoxic activity against four cancer cell lines SF-268, MCF-7, HepG2 and A549 with IC_{50} values of 10.28 ± 0.14-19.11 ± 2.23 μM.	[9]
**33**	Spiroindimicin H				Cytotoxic activity against four cancer cell lines SF-268, MCF-7, HepG2 and A549 with IC_{50} values of 18.16 ± 0.59-33.02 ± 3.41 μM.	
**34**	Indimicin F				-	
**35**	Indimicin G				-	
**36**	Spiroindimicin E	Halogenated bisindole	*Streptomyces* sp. MP131-18	A deep-water marine sediment sample collected in the Trondheim fjord, Norway	Weak cytotoxic activity against T24 bladder carcinoma cells	[10]
**37**	Spiroindimicin F				-	
**38**	dionemycin	Halogenated bisindole	*Streptomyces* sp. SCSIO 11791	A sediment sample collected from the South China Sea	Cytotoxic activity against six cancer cell lines MDA-MB-435, MDA-MB-231, NCI-H460, HCT-116, HepG2 and MCF10A with IC_{50} values of 3.1–	[11]

					11.2 μM. Antimicrobial activity against *M. luteus* and *S. aureus* with MIC values of 0.5 and 1 μM, respectively.	
**39**	6-OMe-7′,7″-dichorochromopyrrolic acid				Cytotoxic activity against four cancer cell lines MDA-MB-435, HCT-116, HepG-2 and MCF-10A with IC_{50} values of 2.9–19.4 μM. Antimicrobial activity against *M. luteus* and *S. aureus* with MIC values of 16 and 3 μM, respectively.	
**40**	Xiamycin C	Indole sesquiterpenoid	*Streptomyces* sp. HK-18	A saltern sediment sample was collected on Shinui Island (445-9, Sangtaeseo-ri, Shinui-myeon, Shinan-gun, Jollanam-do) in the Republic of Korea.	Antiviral activity against porcine epidemic diarrhea virus (PEDV) with an EC_{50} value of 11.49 ± 1.36 μM.	[12]
**41**	Xiamycin D				Antiviral activity against porcine epidemic diarrhea virus (PEDV) with an EC_{50} value of 0.93 ± 0.07 μM.	
**42**	Xiamycin E				Antiviral activity against porcine epidemic diarrhea virus (PEDV) with an EC_{50} value of 2.89 ± 0.36 μM.	
**43**	Lipoxiamycin A	Indole sesquiterpenoid	*Streptomyces* sp. HK-18	A saltern sediment sample was collected on Shinui Island (445-9, Sangtaeseo-ri, Shinui-myeon, Shinan-gun, Jollanam-do) in the Republic of Korea.	Moderately inhibited the production of lipopolysaccharide-induced NO with an IC_{50} value of 9.89 ± 0.92 μM.	[13]
**44**	Lipoxiamycin B				-	
**45**	Dixiamycin A				Significantly inhibited the production of lipopolysaccharide-induced NO with an IC_{50} value of 4.12 ± 0.22 μM.	
**46**	Dixiamycin C				-	
**47**	Pratensilin A	Indolinone-naphthofuran	*Streptomyces pratensis* KCB-132	A marine sediment collected off the Bohai Sea, China	Cytotoxic activity against six cancer cell lines NCI-H1975, NCI-H460, JHH-7, C6, HepG2 and THP-1 with IC_{50} values of 18.4-67.4μM.	[14]
**48**	Pratensilin B				-	
**49**	Pratensilin C				-	

**50**	Pratensilin D	Indolinone-naphthofuran	*Streptomyces pratensis* KCB-132	A marine sediment collected off the Bohai Sea, China	(-)-**51** exhibited antibacterial activity against *B. cereus* with an MIC value of 4 μg/mL and cytotoxic effect on NCI-H460 and HepG2 cell lines with IC_{50} values of 4.6 and 9.3 μg/mL, respectively.	[15]
**51**	Anthranoside C	Miscellaneous indole	*Streptomyces* sp. CMN-62	An unidentified sponge sample collected from NaoZhou Island of the Guangdong Province, China	Antiviral activity against influenza A H_1N_1 virus with an IC_{50} value of 171 μM.	[16]
**52**	Streptoindole A	Miscellaneous indole	*Streptomyces* sp. ZZ1118	A gut sample of marine shrimp (*Penaeus* sp.) obtained from fishing vessel operating in East China Sea, close to Zhoushan archipelago, Zhejiang, China	Antimicrobial activity against MRSA, *E. coli* and *C. albicans* with MIC values of 13–25 μg/mL.	[17]
**53**	Streptoindole B				Antimicrobial activity against MRSA, *E. coli* and *C. albicans* with MIC values of 7–25 μg/mL.	
**54**	Streptoindole C				Antimicrobial activity against *E. coli* and *C. albicans* with both MIC values of 7 μg/mL.	
**55**	Streptoindole D				Antimicrobial activity against MRSA with a MIC value of 25 μg/mL.	
**56**	tirandamycin K	Pyrrolidone	*Streptomyces* sp. 307–9 *ΔtamI*	Marine-derived	-	[18]
**57**	Isotirandamycin B	Pyrrolidone	*Streptomyces* sp. SCSIO 41399	The *Porites* sp. coral collected from the Wenchang, Hainan province of China	Antimicrobial activity against *S. agalactiae* with a MIC value of 5.0 μg/mL.	[19]
**58**	Tirandamycin A				Antimicrobial activity against *S. agalactiae* with a MIC value of 2.5 μg/mL.	
**59**	Tirandamycin B				Antimicrobial activity against *S. agalactiae* with a MIC value of 2.5 μg/mL.	
**60**	Thiolopyrrolone A	Pyrrolone	*Streptomyces* sp. BTBU20218885	A mud sample collected from the intertidal zone, Xiamen, China	Antimicrobial activity against BCG, *M. tuberculosis* and *S. aureus* with MIC values of 10, 10 and 100 μg/mL, respectively.	[20]

**61**	2,2-dioxidothiolutin				Antimicrobial activity against *S. aureus* with a MIC value of 50 μg/mL.	
**62**	Bacillimide	Pyrrolone	*Streptomyces bacillaris* MBTC38	Underwater sediment samples on Jeju Island	Enzyme inhibition against *C. albicans* isocitrate lyase with an IC_{50} value of 44.24 μM.	[21]
**63**	Salinazinone A	Pyrrolidinyl-oxazinone	*Streptomyces* sp. KMF-004	A solar saltern at Aphae Island in Korea	-	[22]
**64**	Salinazinone B				An inhibitory effect on LPS-induced NO production by BV-2 microglia cells with an IC_{50} value of 17.7 μM.	
**66**	Anandin A	Pyrrolidone	*Streptomyces anandii* H41-59	A sea sediment sample from the mangrove zone in the South China Sea	Cytotoxic activity against three cancer cell lines MCF-7, SF-268 and NCI-H460 with IC_{50} values of 7.5, 7.9 and 7.8 μg/mL, respectively.	[23]
**67**	Anandin B				-	
**68**	Ligiamycin A	pyrrolidone	Co-culture of *Streptomyces* sp.GET02.ST and *Achromobacter* sp. GET02.AC	A gut sample of the wharf roaches *Ligia exotica* from the intertidal zone of the west coast of Korea	Antimicrobial activity against *S. aureus* and *S. enterica* with both MIC values of 16 μg/mL.<br>Cytotoxic activity against A549 cell line with an IC_{50} value of 42.0 μM.	[24]
**69**	Ligiamycin B				Antimicrobial activity against *S. aureus* with a MIC value of 64 μg/mL.<br>Cytotoxic activity against five cancer cell lines SNU638, SK-HEP-1, A549, HCT-116 and MDA-MB-23 with IC_{50} values of 20.1-46.2 μM.	
**70**	Oxoprothracarcin	pyrrolobenzodiazepine	*Streptomyces* sp. M10946	Mangrove sediment collected from Hut Bay	Antiproliferative effects against MDA-MB-231 cells and A549 cells at a concentration of 10 μM with growth inhibition rates of 10.2% and 7.3%	[25]
**71**	7-methoxy-8-hydroxy cycloanthranilylproline	pyrrolobenzodiazepine	*Streptomyces cacaoi* 14CM034	The sediments that collected from Mersin-Turkey at a depth of 8 m Mediterranean Sea	Antimicrobial activity against *E. coli*, MRSA, *E. faecium*, *P.aeruginosa* and *C. albicans* with MIC values of 8.75-17.5 μg/mL.	[26]
**72**	Cycloanthranilylproline				Antimicrobial activity against *E. coli*, MRSA, *E. faecium*, *P.aeruginosa* and *C. albicans* with MIC	

					values of 16-32 μg/mL.	
**73**	Hygrocin C	Ansamycin (Pyrrole)	*Streptomyces* sp. LZ35ΔGPKS	Intertidal soil collected at Jimei, Xiamen, China	Cytotoxic activity against MDA-MB-431 and PC3 cell lines with IC_{50} values of 0.5 and 1.9 μg/mL, respectively.	[27]
**74**	Hygrocin D				Cytotoxic activity against MDA-MB-431 and PC3 cell lines with IC_{50} values of 3.0 and 5.0 μg/mL, respectively.	
**75**	Hygrocin E				-	
**76**	Hygrocin F				Cytotoxic activity against MDA-MB-431 and PC3 cell lines with IC_{50} values of 3.3 and 4.5 μg/mL, respectively.	
**77**	Hygrocin H	Ansamycin (Pyrrole)	*Streptomyces* sp. LZ35 mutant strain SR101OEhgc1	Intertidal soil collected at Jimei, Xiamen, China	Cytotoxic activity against MDA-MB-231, PC3 and HeLa cell lines with IC_{50} values of 2.4, 1.7, and 0.8 μM, respectively.	[28]
**78**	Hygrocin I				-	
**79**	Hygrocin G				-	
**80**	Hygrocin K	Ansamycin (Pyrrole)	*Streptomyces* sp. ZZ1956	A marine mud sample collected from the mangrove area (4.15° S, 119.61° E) of Pangkep District South Sulawesi Province, Indonesia	-	[29]
**81**	Hygrocin L				-	
**82**	Hygrocin M				-	
**83**	Hygrocin N				Cytotoxic activity against U87MG and U251 cell lines with IC_{50} values of 8.17 ± 0.17 and 7.04 ± 0.28 μM, respectively. Antimicrobial activity against MRSA and *E. coli* with MIC values of 15 and 8 μg/mL, respectively.	
**84**	Hygrocin O				Antimicrobial activity against MRSA and *E. coli* with MIC values of 24 and 20 μg/mL, respectively.	
**85**	Hygrocin P				-	

**86**	Hygrocin Q				Cytotoxic activity against U87MG and U251 cell lines with IC_{50} values of 8.81 ± 0.80 and 10.46 ± 0.27 μM, respectively.	
**87**	Hygrocin R				Cytotoxic activity against U87MG and U251 cell lines with IC_{50} values of 8.32 ± 0.38 and 7.86 ± 0.26 μM, respectively.<br>Antimicrobial activity against MRSA and *E. coli* with MIC values of 9 and 16 μg/mL, respectively.	
**88**	Hygrocin S				-	
**89**	Ansalactam B	Ansamycin (Pyrrole)	*Streptomycetes* sp. CNH189	Aangrove sediment sample, Danzhou, China	Weak antimicrobial activity against MRSA with a MIC value of 31.2 μg/mL.	[30]
**90**	Ansalactam C				Weak antimicrobial activity against MRSA with a MIC value of 31.2 μg/mL.	
**91**	Ansalactam D				Weak antimicrobial activity against MRSA with a MIC value of 62.5 μg/mL.	
**92**	Divergolide T	Ansamycin (Pyrrole)	*Streptomyces* sp. KFD18	Mangrove sediment, collected from Danzhou, Hainan province in China	Cytotoxic activity against human cancer cell lines SGC-7901, K562, Hela and A549 with IC_{50} values of 2.8-14.9 μM.	[31]
**93**	Divergolide U				Cytotoxic activity against human cancer cell lines SGC-7901, K562 and A549 with IC_{50} values of 9.0-24.7 μM.	
**94**	Divergolide V				Cytotoxic activity against human cancer cell lines SGC-7901, K562, Hela and A549 with IC_{50} values of 4.7-20.9 μM.	
**95**	Divergolide W				Cytotoxic activity against human cancer cell lines SGC-7901, K562, Hela and A549 with IC_{50} values of 16.3-33.2 μM.	
**96**	Chlorizidine A	Miscellaneous pyrrole	*Streptomyces* sp. CNH-287	Marine-derived	Cytotoxic activity against HCT-116 cell line with IC_{50} values of 3.2-4.9 μM.	[32]

**97**	Streptopyrrole B	Miscellaneous pyrrole	*Streptomyces zhaozhouensis* 208DD-064	Marine sediment samples collected offshore of Dokdo Island, Republic of Korea	Antimicrobial activity against *B. subtilis*, *M. luteus* and *S. aureus* with all MIC values of 0.8 μM. Cytotoxic activity against six cancer cell lines PC-3, NCI-H23, HCT-15, NUGC-3, ACHN and MDA-MB-231 with IC_{50} values of 4.9-6.6 μM.	[33]
**98**	Streptopyrrole C				Antimicrobial activity against *B. subtilis*, *M. luteus* and *S. aureus* with MIC values of 0.7-2.9 μM.	
**99**	16-deethylindanomycin	Miscellaneous pyrrole	*Streptomyces antibioticus* PTZ0016	Marine sediments	Antimicrobial activity against *S. aureus* with a MIC value of 4.0 μM.	[34]
**100**	iso-16-deethylindanomycin				Antimicrobial activity against *S. aureus* with a MIC value of 6.0 μM.	
**101**	16-deethylindanomycin methyl ester				Antimicrobial activity against *S. aureus* with a MIC value of 6.0 μM.	
**102**	iso-16-deethylindanomycin methyl ester				Antimicrobial activity against *S. aureus* with a MIC value of 8.0 μM.	
**103**	Nitricquinomycin A	Miscellaneous pyrrole	*Streptomyces* sp. ZS-A45	Marine sediments of Zhou Shan Island (29°56′ N, 122°16′ E) Zhejing province, China	-	[35]
**104**	Nitricquinomycin B				-	
**105**	Nitricquinomycin C				Cytotoxic activity against PC-3 and A2780 cell lines with IC_{50} values of 17.28 ± 0.60 and 4.77 ± 0.03 μM, respectively. Antimicrobial activity against *E. coli, S. aureus and C. albicans* with MIC values of 20-40 μM.	
**106**	Bhimamycin J	Miscellaneous pyrrole	*Streptomyces* sp. MS180069	A sediment sample collected by gravity corer from the South China Sea	Enzyme inhibition against ACE2 with inhibition rate of 70% at 25 μg/mL.	[36]
**107**	Glaciapyrrole D	Miscellaneous pyrrole	*Streptomyces* sp. GGS53	A deep-sea sediment core collected in the East Sea of	-	[37]

**108**	Glaciapyrrole E			Korea (37°12.0486′, 130°25.0889′, ca. 2163 m in water depth)	Significant antiviral activity and resulted in the reduction of the viral titer by 70%.	
**109**	Pulicatin J	oxazole and thiazole	*Streptomyces diastaticus* NBU2966	A marine sponge *Axinellida* sp. at 76 m depth in the Mesophotic Coral Ecosystems located near Apo Island, Negros Oriental, Philippines (9°04′33.0″ N, 123°15′59.1″ E)	-	[38]
**110**	Thiazostatin C				-	
**111**	Thiazostatin B				-	
**112**	Spoxazomicin E				-	
**113**	Streptochelin A				-	
**114**	Piericidin F	Piericidin (Pyridine)	*Streptomyces* sp. CHQ-64 Δ*rdmF*	Reed rhizosphere soil collected from the mangrove conservation area of Guangdong Province, China.	Cytotoxic activity against four cancer cell lines HeLa, NB4, A549 and H1975 with IC_{50} values of 0.003-0.56 μM.	[39]
**115**	11-demethyl-piericidin A	Piericidin (Pyridine)	*Streptomyces psammoticus* SCSIO NS126	A mangrove sediment sample collected from the Pearl River estuary (E 113°33′11.15″, N 22°53′40.16″) to South China Sea	Cytotoxic activity against two renal carcinoma cell lines ACHN and OS-RC-2 with IC_{50} values of 4.1 and 11 μM, respectively.	[40]
**116**	(2*E*,5*E*,7*E*,11*E*,9*R*,10*R*,13*S*,19*R*)-13,19-Dihydroxyl-IT-143-A				Cytotoxic activity against two renal carcinoma cell lines ACHN and OS-RC-2 with IC_{50} values of 2.4 and 5.3 μM, respectively.	
**117**	(2*E*,5*E*,7*E*,11*E*,9*R*,10*R*,13*R*,19*S*)-13,19-Dihydroxyl-IT-143-A				Cytotoxic activity against two renal carcinoma cell lines ACHN and OS-RC-2 with IC_{50} values of 3.8 and 4.1 μM, respectively.	
**118**	10-Ketone piericidin A				Cytotoxic activity against renal carcinoma cell line OS-RC-2 with an IC_{50} value of 22 μM.	
**119**	7-Demethylglucopiericidin A				Cytotoxic activity against three renal carcinoma cell lines ACHN, OS-RC-2 and 786-O with IC_{50} values of 0.31, 2.6 and 0.99 μM, respectively.	
**120**	7-Demethyl-13-hydroxyglucopiericidin A				Cytotoxic activity against three renal carcinoma cell lines ACHN, OS-RC-2 and 786-O with IC_{50}	

					values of 2.5, 79 and 28 μM, respectively.	
**121**	13-Hydroxypiericidin A 10-*O*-*α*-D-galactose (1→6)-*β*-D-glucoside				-	
**122**	13-Hydroxypiericidin A 10-*O*-*α*-D--glucose (1→6)-*β*-D-glucoside				Cytotoxic activity against renal carcinoma cell line ACHN with an IC_{50} value of 23 μM.	
**123**	4′-*O*-*β*-D-Glucose glucopiericidin A				Cytotoxic activity against renal carcinoma cell line ACHN with an IC_{50} value of 28 μM.	
**124**	4′-*O*-*β*-D-Glucose 13-hydroxyglucopiericidin A				-	
**125**	4′-*O*-*β*-D-Glucose piericidin A 10-*O*-*α*-D-glucose (1→6)-*β*-D-glucoside				-	
**126**	5-Hydroxy-6-hydroxymethyl glucopiericidin A				Cytotoxic activity against renal carcinoma cell line ACHN with an IC_{50} value of 2.4 μM.	
**127**	5-Hydroxy-6-hydroxymethyl-13-hydroxyglucopiericidin A				Cytotoxic activity against renal carcinoma cell line ACHN with an IC_{50} value of 60 μM.	
**128**	2-Hydroxymethyl-Δ3, 4-glucopiericidin A				Cytotoxic activity against three renal carcinoma cell lines ACHN, OS-RC-2 and 786-O with IC_{50} values of 1.7, 60 and 28 μM, respectively.	
**129**	(11*S*,12*R*)Piericidin C1 10-*O*-*β*-D-glucoside				Cytotoxic activity against three renal carcinoma cell lines ACHN, OS-RC-2 and 786-O with IC_{50} values of 2.8, 30 and 19 μM, respectively.	
**130**	(11*R*,12*S*)Piericidin C1 10-*O*-*β*-*D*-glucoside				-	
**131**	13-Dimethoxy glucopiericidin A				-	

**132**	Piericidin A5	Piericidin (Pyridine)	*Streptomyces* sp. SCSIO 40063	A sediment sample (E 111° 41′, N 16°33′) at the depth of 10 m collected from Yagong island of Sansha City, Hainan Province, China	-	[41]
**133**	Piericidin G1				Cytotoxic activity against four cancer cell lines SF-268, MCF-7, HepG2 and A549 with IC_{50} values of 10.0-12.7 μM.	
**134**	Actinoquinoline A	Quinoline	*Streptomyces* sp. CNP975	A marine sediment sample collected in La Jolla, CA, USA by Alejandra Prieto Davó	Inhibition of COX-1 and COX-2 with IC_{50} values of 7.6 and 2.13 μM, respectively.	[42]
**135**	Actinoquinoline B				Inhibition of COX-1 and COX-2 with IC_{50} values of 4.9 and 1.42 μM, respectively.	
**136**	Unnamed	Quinoline	*Streptomyces cyaneofuscatus* M-157	A solitary coral belonging to the order Scleractinia collected at 2000 m depth in Cantabrian Sea, Biscay Bay, Northeast Atlantic	Weak cytotoxic activity against HepG2 cell line with an IC_{50} value of 51.5 μM.	[43]
**137**	Unnamed				-	
**138**	Unnamed				-	
**139**	Diazaquinomycin E	Diaza-anthracene	*Streptomyces* sp. F001	Marine-derived	Cytotoxic activity against the ovarian cancer cell line OVCAR5 with an IC_{50} value of 9.0 μM.	[44]
**140**	Diazaquinomycin F				-	
**141**	Diazaquinomycin G				-	
**142**	Ageloline A	Quinoline	*Streptomyces* sp. SBT345	Mediterranean sponge *Agelas oroides*	Inhibition of *Chlamydia trachomatis* growth with an IC_{50} value of 9.54 ± 0.36 μM by inhibiting reactive ROS production during the early stages of infection.	[45]
**143**	Mansouramycin E	Isoquinolinequinone	*Streptomyces* sp. B1848	The Actinomycetes culture collection of the Alfred-Wegner Institute for Polar-und Marine Research, Am Handelshafen, Bremen,	Cytotoxic activity against 36 cancer cell lines with mean IC_{50} values of 23.10 μM.	[46]
**144**	Mansouramycin F				Cytotoxic activity against 36 cancer cell lines with mean IC_{50} values of 7.92 μM.	

**145**	Mansouramycin G			Germany	-	
**146**	Strepchazolin A	Miscellaneous pyridine	*Streptomyces chartreusis* NA02069	A sediment from the coast of Hainan Island, China.	Antimicrobial activity against *B. subtilis* with a MIC value of 64.0 μM.<br>Enzyme inhibition against AChE with an IC_{50} value of 50.6 μM.	[47]
**147**	Strepchazolin B				-	
**148**	Streptoglutarimide A	Miscellaneous pyridine	*Streptomyces* sp. ZZ741	A sample (HQ-2) of marine mud collected from the coastal area of Jintang Island (30°06′39″ N and 121°48′07″ E) in Zhoushan, China	Antimicrobial activity against MRSA, *E. coli* and *C. albicans* with MIC values of 9, 9 and 16 μM, respectively.	[48]
**149**	Streptoglutarimide B				Antimicrobial activity against MRSA, *E. coli* and *C. albicans* with MIC values of 11, 9 and 20 μM, respectively.	
**150**	Streptoglutarimide C				Antimicrobial activity against MRSA, *E. coli* and *C. albicans* with MIC values of 10, 8 and 15 μM, respectively.	
**151**	Streptoglutarimide D				Antimicrobial activity against MRSA, *E. coli* and *C. albicans* with MIC values of 10, 8 and 8 μM, respectively.	
**152**	Streptoglutarimide E				Antimicrobial activity against MRSA, *E. coli* and *C. albicans* with MIC values of 9, 10 and 20 μM, respectively.	
**153**	Streptoglutarimide F				Antimicrobial activity against MRSA, *E. coli* and *C. albicans* with MIC values of 10, 12 and 18 μM, respectively.	
**154**	Streptoglutarimide G				Antimicrobial activity against MRSA, *E. coli* and *C. albicans* with MIC values of 10, 12 and 18 μM, respectively.	
**155**	Streptoglutarimide H				Antimicrobial activity against MRSA, *E. coli* and *C. albicans* with MIC values of 9, 10 and 15 μM, respectively.<br>Cytotoxic activity against human glioma U87MG and U251 cell lines with IC_{50} values of 3.8 ± 0.6 and	

					1.5 ± 0.1 μM, respectively.	
**156**	Streptoglutarimide I				Antimicrobial activity against MRSA, *E. coli* and *C. albicans* with MIC values of 11, 9 and 12 μM, respectively.	
**157**	Streptoglutarimide J				Antimicrobial activity against MRSA, *E. coli* and *C. albicans* with MIC values of 10, 10 and 18 μM, respectively.	
**158**	Griseusrazin A	Pyrazine	*Streptomyces griseus* subsp. *griseus* 09-0144	Tidal flat sediments on Hwangdo in the Yellow Sea, South Korea	Dose-dependently inhibition the production of NO and PGE_2 and the iNOS and COX-2 protein expression.	[49]
**159**	(*S*)-6-(*sec*-butyl)-3-isopropylpyrazin-2(1*H*)-one	Pyrazinone	*Streptomyces* sp. Did-27	The marine tunicate *Didemnum* sp. in Obhur, Saudi Arabia	Cytotoxic activity against HCT-116 and MCF-7 cell lines with IC_{50} values of 30 and 25 μM, respectively.	[50]
**160**	(*S*)-6-(*sec*-butyl)-6-isopropylpyrazin-2(1*H*)-one				-	
**161**	(*S*)-6-(*sec*-butyl)-3--isobutylpyrazin-2(1*H*)-one				Cytotoxic activity against HCT-116 and MCF-7 cell lines with IC_{50} values of 30 and 35 μM, respectively.	
**162**	Streptopyrazinone A	Pyrazinone	*Streptomyces* sp. ZZ446	A sample of coastal soil, which was collected from Zhoushan Islands, Zhejiang, China	Antimicrobial activity against MRSA and *C. albicans* with MIC values of 58.0 and 35.0 μM, respectively.	[51]
**163**	Streptopyrazinone B				Antimicrobial activity against MRSA and *C. albicans* with MIC values of 60.0 and 38.0 μM, respectively.	
**164**	Streptopyrazinone C				Antimicrobial activity against MRSA and *C. albicans* with MIC values of 62.0 and 45.0 μM, respectively.	
**165**	Streptopyrazinone D				Antimicrobial activity against MRSA and *C. albicans* with MIC values of 65.0 and 38.0 μM, respectively.	
**166**	Actinopolymorphol E	Pyrazine	*Streptomyces* sp.	The marine sediment	-	[52]

**167**	Actinopolymorphol F		CNP-944	collected at the La Jolla Submarine Canyon, California	Antimicrobial activity against *K. rhizophila*, *B. subtilis* and *S. aureus* with MIC values of 16-64 µg/mL.	
**168**	3-(3-hydroxy-4-methoxybenzyl)-6isobutyl-2,5-diketopiperazine	2,5-diketopiperazine	*Streptomyces* sp. MNU FJ-36	The intestinal fabric of *Katsuwonus* sp.	Cytotoxic activity against A-549 cell line with an IC_{50} value of 89.4 ± 5 µg/mL.	[53]
**169**	3-(1,3-benzodioxol-5-ylmethyl)-6isobutyl-2,5-diketopiperazine				Cytotoxic activity against HCT-116 and A-549 cell lines with IC_{50} values of 75.4 ± 4 and 35.4 ± 7 µg/mL, respectively.	
**170**	3-(1,3-benzodioxol-5-ylmethyl)6-isopropyl-2,5-diketopiperazine				Cytotoxic activity against HCT-116 and A-549 cell lines with IC_{50} values of 45.4 ± 6 and 28.4 ± 5 µg/mL, respectively.	
**171**	Streptodiketopiperazine A	2,5-diketopiperazine	*Streptomyces* sp. SY1965	Hadal sediments MTD11000, which was collected from the Mariana Trench (11°20′ N and 142°11.5′ E) at depth 11,000 m	Antimicrobial activity against *C. albicans* with a MIC value of 42 µg/mL.	[54]
**172**	Streptodiketopiperazine B				Antimicrobial activity against *C. albicans* with a MIC value of 42 µg/mL.	
**173**	Naseseazine C	2,5-diketopiperazine	*Streptomyces* sp. USC-636	Marine sediment obtained on the Sunshine Coast, QLD, Australia	Inhibition of chloroquine-sensitive *Plasmodium falciparum* with an IC_{50} value of 3.52 ± 1.2 µM.	[55]
**174**	Actinozine A	2,5-diketopiperazine	*Streptomyces* sp. Call-36	The Red Sea sponge *Callyspongia* species	Antimicrobial activity against *S. aureus* and *C. albicans* with inhibition zones of 23.0 and 19.0 mm, respectively.	[56]
**175**	Cyclo(2-OH-D-Pro-L-Leu)				Antimicrobial activity against *S. aureus* and *C. albicans* with inhibition zones of 20.0 and 16.0 mm, respectively.	
**176**	Maculosin-*O*-α-L-rhamnopyranoside	2,5-diketopiperazine	*Streptomyces* sp. ZZ446	Coastal soil	Antimicrobial activity against MRSA, *E. coll* and *C. albican* with MIC values of 26.0-37.0 µg/mL.	[57]
**177**	Maculosin				Antimicrobial activity against MRSA, *E. coli* and *C. albicans* with MIC values of 27.0-37.0 µg/mL.	
**178**	Venezueline A	Phenoxazine	*Streptomyces*	The ocean sediment near	-	[58]

**179**	Venezueline B		*venezuelae* KHG20-22	Guam Island (Pacific Ocean)	Cytotoxic activity against five cancer cell lines HCT-8, BGC-823, A549, A2780 and NIH-H460 with IC_{50} values of 5.74-9.67 μM.	
**180**	Venezueline C				-	
**181**	Venezueline D				-	
**182**	Venezueline E				-	
**183**	Maroxazinone	Phenoxazine	*Streptomyces* sp. Eg25	A sea sand sample collected from Marsa Matruh city (Egypt)	Cytotoxic activity against three cancer cell lines MCF-7, HEPG-2 and HCT-116 with IC_{50} values of 4.32, 2.90 and 8.51 μg/mL, respectively.	[59]
**184**	Streptophenazine I	phenazine	*Streptomyces* sp. HB202	The marine breadcrumb sponge *Halichondria panicea* (class Demospongia) collected from the Baltic Sea, Germany	Enzyme inhibition against enzyme phosphodiesterase with an IC_{50} value of 11.6 ± 1.1 μM.	[60]
**185**	Streptophenazine J				Enzyme inhibition against enzyme phosphodiesterase with an IC_{50} value of 2.0 ± 0.9 μM.	
**186**	Streptophenazine K				Enzyme inhibition against enzyme phosphodiesterase with an IC_{50} value of 12.2 ± 2.0 μM.<br>Antimicrobial activity against *B. subtilis* and *S. epidermidis* with MIC values of 21.6 ± 6.8 μM and 14.5 ± 2.0 μM, respectively.	
**187**	Strepoxazine A	phenoxazine	*Streptomyces* sp. SBT345	The Mediterranean sponge *Agelas oroides*	Cytotoxic activity against human promyelocytic leukemia cells HL-60 with an IC_{50} value of 16 μg/mL.	[61]
**188**	Neo-actinomycin A	phenoxazine	*Streptomyces* sp. IMB094	A marine sediment sample collected at a depth of ca. 40 m from Heishijiao Bay, Dalian, China	Antimicrobial activity against MRSA and VRE with MIC values of 16-64 μg/mL.<br>Cytotoxic activity against HCT-116 and A549 cancer cell lines with IC_{50} values of 38.7 nM and 65.8 nM, respectively.	[62]
**189**	Neo-actinomycin B				-	

**191**	Actimomycin S	phenoxazine	*Streptomyces* sp. S22	A sediment sample collected from a saline-alkali land in the Huanghe River delta, China	Antimicrobial activity against pathogenic bacteria *E. faecium*, *S. aureus*, *K. pneumoniae*, *A. baumannii* and *E. coli* with all MIC values of 5 µg/mL.<br>Cytotoxic activity against HepG2 liver carcinoma cell line with an IC_{50} value of 0.10 µM.	[63]
**192**	Neo-actinomycin C				Antimicrobial activity against *E. faecium*, *S. aureus*, *K. pneumoniae*, *A. baumannii* and *E. coli* with MIC values of 10-80 µg/mL.<br>Cytotoxic activity against HepG2 liver carcinoma cell line with an IC_{50} value of 0.32 µM.	
**193**	Neo-actinomycin D				Antimicrobial activity against *E. faecium*, *S. aureus*, *K. pneumoniae*, *A. baumannii* and *E. coli* with MIC values of 2.5-5 µg/mL.<br>Cytotoxic activity against HepG2 liver carcinoma cell line with an IC_{50} value of 0.024 µM.	
**194**	Cyclizidine B	Cyclizidine (Indolizidine)	*Streptomyces* sp. HNA39	Marine sediments of Hainan Island (Hainan province, China, N 19° 95′ E 110° 58′)	-	[64]
**195**	Cyclizidine C				Cytotoxic activity against PC-3 and HCT-116 cancer cell lines with IC_{50} values of 0.52 ± 0.03 µM and 8.3 ± 0.1 µM, respectively.<br>Enzyme inhibition against protein kinase ROCK2 with an IC_{50} value of 7.0 ± 0.8 µM.	
**196**	Cyclizidine D				Cytotoxic activity against PC-3 and HCT-116 cancer cell lines with IC_{50} values of 33 ±1 µM and 40 ± 1 µM, respectively.	
**197**	Cyclizidine E				-	
**198**	Cyclizidine F				Enzyme inhibition against protein kinase ROCK2 with an IC_{50} value of 27 ± 1 µM.	
**199**	Cyclizidine G				-	
**200**	Cyclizidine H				Cytotoxic activity against PC-3 cancer cell line with an IC_{50} value of 17 ± 1 µM.<br>Enzyme inhibition against protein kinase ROCK2	

					with an IC_{50} value of 42 ± 3 μM.	
**201**	Cyclizidine I				Enzyme inhibition against protein kinase ROCK2 with an IC_{50} value of 39 ± 1 μM.	
**202**	Cyclizidine J	Cyclizidine (Indolizidine)	*Streptomyces* sp. HNA39	Marine sediments of Hainan Island (Hainan province, China, N 19° 95′ E 110° 58′)	-	[65]
**203**	Unnamed	Cyclizidine (piperidine)	*Streptomyces* sp. WU20	Hydrothermal vent sediment, collected from Kueishantao, Taiwan.	Antimicrobial activity against *B. subtilis* with a MIC value of around 32 μg/mL.	[66]
**204**	Streptopertusacin A	Indolizinium	*Streptomyces* sp. HZP-2216E	The fresh sea lettuce Ulva pertusa collected from the Turtle Islet located at 22.46° N and 115.21 ° E in South China Sea close to Shanwei City (Guangdong, China)	Antimicrobial activity against MRSA with a MIC value of 40 mg/mL.	[67]
**205**	Spithioneine A	Pyrrolizidine	*Streptomyces spinoverrucosus* SNB-048	A sand sample collected from a Bahamian tidal flat	-	[68]
**206**	Spithioneine B				-	
**207**	Bohemamine D	Pyrrolizidine	*Streptomyces spinoverrucosus* SNB-048	A sand sample collected from a Bahamian tidal flat	-	[69]
**208**	Bohemamine E				-	
**209**	Bohemamine F				-	
**210**	Bohemamine G				-	
**211**	Bohemamine H				-	
**212**	Bohemamine I				-	
**213**	5-Br-bohemamine C	Pyrrolizidine	*Streptomyces*	A sediment sample collected	-	[70]

**214**	Dibohemamine A		*spinoverrucosus* SNB-032	from amudflat on a small Bahamas island	-	
**215**	Dibohemamine B				Cytotoxic activity against NSCLC cell lines A549 and HCC1171 with IC_{50} values of 0.140 and 3.9 μM, respectively.	
**216**	Dibohemamine C				Cytotoxic activity against NSCLC cell lines A549, HCC44, HCC1171 and HCC366 with IC_{50} values of 0.145-12.0 μM.	
**217**	Carpatamide A	Linear amide	*Streptomyces* sp. SNE-011	A marine sediment sample collected from South Carolina (32°35′10″ N, 80°07′31″ W)	Cytotoxic activity against three cancer cell lines HCC366, A549 and HCC44 with IC_{50} values of 2.8-8.4 μM.	[71]
**218**	Carpatamide B				-	
**219**	Carpatamide C				Cytotoxic activity against three cancer cell lines HCC366 and A549 with IC_{50} values of 2.2 and 3.7 μM, respectively.	
**220**	Antimycin E	Linear amide	*Streptomyces* sp. THS-55	The sediment of mangrove, Sanya, China	Significant cytotoxic activity against HeLa cell line with IC_{50} < 0.1 μM.	[72]
**221**	Antimycin F					
**222**	Antimycin G					
**223**	Antimycin H					
**224**	Neoantimycin A	Linear amide	*Streptomyces antibioticus* H12-15	A marine sediment sample collected at the Taishan mangrove site, Guangdong province, China	Cytotoxic activity against SF-268 cell line with an IC_{50} value of 33.6 μM.	[73]
**225**	Neoantimycin B				Cytotoxic activity against SF-268 cell line with an IC_{50} value of 41.6 μM.	
**226**	Bagremycin C	Linear amide	*Streptomyces* sp. Q22	A sample of mangrove soils obtained from the Qiao Mangrove Forest in Zhuhai City, Guangdong, China	Cytotoxic activity against four cancer cell lines U87MG, U251, SHG44, and C6 with IC_{50} values of 2.2 ± 0.1-6.4 ± 0.5 μM. (inhibited the G_0/G_1 cell cycle)	[74]
**227**	Bagremycin D				-	
**228**	Bagremycin F	Linear amide	*Streptomyces* sp. ZZ745	A sample of marine mud collected from a coastal area	Antimicrobial activity against *E. coli* with a MIC value of 41.8 μg/mL.	[75]

**229**	Bagremycin G			located at the Jintang Island of Zhoushan, Zhejiang, China	Antimicrobial activity against *E. coli* with a MIC value of 67.1 μg/mL.	
**230**	*N*-acetylborrelidin B	Linear amide	*Streptomyces mutabilis* sp. MII	A sediment sample collected from Hurghada Coast at the Red Sea, Egypt	Antimicrobial activity against *E. coli*, *B. subtilis*, *P. agarici*, *M. luteus* and *S. warneri* with inhibition zones of 7-18 mm.	[76]
**231**	Isoikarugamycin	Polycyclic tetramate macrolactams	*Streptomyces zhaozhouensis* CA-185989	A marine sediment collected off-shore at 2 m depth nearby Utonde, Equatorial Guinea	Antimicrobial activity against MRSA, *C. albicans* and *A. fumigatus* with MIC values of 2-8 μg/mL.	[77]
**232**	28-*N*-methylikaguramycin				Antimicrobial activity against MRSA, *C. albicans* and *A. fumigatu* with MIC values of 1-8 μg/mL.	
**233**	30-oxo-28-*N*-methylikarugamycin				Antimicrobial activity against MRSA with MIC values of 32-64 μg/mL.	
**234**	Pactamide A	Polycyclic tetramate macrolactams	*Streptomyces pactum* SCSIO 02999	Deep-sea derived	Cytotoxic activity against cancer cell lines SF-268, MCF-7, NCI-H460 and HepG2 with IC_{50} values of 0.24 ± 0.02-0.51 ± 0.01 μM.	[78]
**235**	Pactamide B				Cytotoxic activity against cancer cell lines SF-268, MCF-7, NCI-H460 and HepG2 with IC_{50} values of 21.93 ± 0.58-26.15 ± 1.37 μM.	
**236**	Pactamide C				Cytotoxic activity against SF-268, MCF-7, NCI-H460 and HepG2 with IC_{50} values of 0.71 ± 0.02-2.42 ± 0.15 μM.	
**237**	Pactamide D				Cytotoxic activity against SF-268, MCF-7, NCI-H460 and HepG2 with IC_{50} values of 14.50 ± 0.02-2.42 ± 0.15 μM.	
**238**	Pactamide E				Cytotoxic activity against SF-268, MCF-7, NCI-H460 and HepG2 with IC_{50} values of 5.10 ± 1.43-8.70 ± 0.25 μM.	
**239**	Pactamide F				Cytotoxic activity against SF-268, MCF-7, NCI-H460 and HepG2 with IC_{50} values of 2.65 ± 0.29-2.85 ± 0.22 μM.	
**240**	Chlokamycin	Polycyclic tetramate macrolactams	*Streptomyces* sp. MA2-12	The Marine Biosciences Kamaishi Research laboratory	Cytotoxic activity against Jurkat and HCT116 cell lines with IC_{50} values of 24.7 μM and 33.5 μM, respectively.	[79]

**241**	10-*epi*-HSAF	Polycyclic tetramate macrolactams	*Streptomyces* sp. SCSIO 40010	The Mangrove sediment obtained from Penang, Malaysia	Cytotoxic activity against SF-268, MCF-7, A549 and HepG2 with IC_{50} values of 2.47 ± 0.05-5.99 ± 0.15 μM.	[80]
**242**	10-*epi*-deOH-HSAF				Cytotoxic activity against SF-268, MCF-7, A549 and HepG2 with IC_{50} values of 3.84 ± 0.07-11.01 ± 1.09 μM.	
**243**	10-*epi*-maltophilin				Cytotoxic activity against SF-268, MCF-7, A549 and HepG2 with IC_{50} values of 3.18 ± 0.13-6.30 ± 0.34 μM.	
**244**	10-*epi*-xanthobaccin C				Cytotoxic activity against SF-268, MCF-7, A549 and HepG2 with IC_{50} values of 3.54 ± 0.24-17.86 ± 0.62 μM.	
**245**	10-*epi*-hydroxymaltophilin				Cytotoxic activity against SF-268, MCF-7, A549 and HepG2 with IC_{50} values of 3.12 ± 0.11-6.83 ± 0.36 μM.	
**246**	10-*epi*-FI-2				-	
**247**	JBIR-150	Macrolactam	*Streptomyces* sp. OPMA00071	Marine sediments collected in Okinawa prefecture, Japan	Cytotoxic activity against Jurkat and MESO-1 cell lines with IC_{50} values of 0.9 μM and 2.3 μM, respectively.	[81]
**248**	Muanlactam	Macrolactam	*Streptomyces* sp. MA159	Intertidal mudflats	Cytotoxic activity against HCT-116 cell line with an IC_{50} value of 1.58 μM.	[82]
**249**	Cyclamenol E	Macrolactam	*Streptomyces* sp. OUCMDZ-4348	Antarctica	Cytotoxic activity against the gastric carcinoma cell line N87 with an IC_{50} value of 9.8 μM.	[83]
**250**	Cyclamenol F				-	
**251**	Somalactam A	Macrolactam	*Streptomyces somaliensis* 1107	An Arctic *Haliclona* sponge	Inhibition of IL-6 and TNF-α release in the serum of LPS-stimulated RAW264.7 macrophage cells with IC_{50} values of 5.76 μM and 0.18 μM, respectively.	[84]
**252**	Somalactam B				-	
**253**	Somalactam C				-	

**254**	Somalactam D				-	
**255**	Niphimycin C	Guanidylpolyol macrolide	*Streptomyces* sp. IMB7-145	A marine sediment sample collected at a depth of ca. 40 m from Heishijiao Bay, Dalian, China	Antimicrobial activity against Gram-positive bacteria *S. epidermidis*, *S. aureus*, *E. faecalis*, *E. faecium* and fungi *C. albicans* with MIC values of 8-64 μg/mL and *Mycobacterium tuberculosis* strains with MIC values of 4-32 μg/mL.<br>Cytotoxic activity against cancer cell lines K562, HepG2, MCF-7 and HeLa with IC_{50} values of 8.5-24 μM.	[85]
**256**	Niphimycin D				Antimicrobial activity against *S. aureu* and fungi *C. albicans* with both MIC values of 64 μg/mL.<br>Cytotoxic activity against HeLa cancer cell line with an IC_{50} value of 22.4 μM.	
**257**	Niphimycin E				Antimicrobial activity against *S. epidermidis*, *S. aureu* and fungi *C. albicans* with MIC values of 16-32 μg/mL.<br>Cytotoxic activity against cancer cell lines K562, HepG2, MCF-7 and HeLa with IC_{50} values of 9-20.8 μM.	
**258**	17-O-methyl-niphimycin				Antimicrobial activity against Gram-positive bacteria *S. epidermidis*, *S. aureus*, *E. faecalis*, *E. faecium* and *C. albicans* with MIC values of 8-64 μg/mL.<br>Cytotoxic activity against cancer cell lines K562, HepG2, MCF-7 and HeLa with IC_{50} values of 3-16.2 μM.	
**259**	Antartin	Sesquiterpene	*Streptomyces* sp. SCO736	Marine sediments collected off the coast of Antarctica	Cytotoxic activity against twelve human cancer cells with GI_{50} of 4-8 μg/mL and inhibited the production of solid lung tumor cells.	[86]
**260**	Penzonemycin A	phenylhydrazone	*Streptomyces* sp. SCSIO 40020	A marine sediment sample (E 114.0432°, N 22.0194°, Pearl River Estuary in China at the depth of 28 m	Cytotoxic activity against cancer cell lines SF-268, MCF-7, A549 and HepG-2 with IC_{50} values of 30.44-61.92 μM.	[87]
**261**	Penzonemycin B				-	

References

1. Cheng, X.; Zhou, B.; Liu, H.; Huo, C.; Ding, W. One new indolocarbazole alkaloid from the *Streptomyces* sp. A22. *Nat. Prod. Res.* **2018,** 32, (21), 2583-2588.
2. Wang, J. N.; Zhang, H. J.; Li, J. Q.; Ding, W. J.; Ma, Z. J. Bioactive indolocarbazoles from the marine-derived *Streptomyces* sp. DT-A61. *J. Nat. Prod.* **2018,** 81, (4), 949-956.
3. Qin, L.; Zhou, B.; Ding, W.; Ma, Z. Bioactive metabolites from marine-derived *Streptomyces* sp. A68 and its Rifampicin resistant mutant strain R-M1. *Phytochem. Lett.* **2018,** 23, 46-51.
4. Zhou, B.; Hu, Z. J.; Zhang, H. J.; Li, J. Q.; Ding, W. J.; Ma, Z. J. Bioactive staurosporine derivatives from the *Streptomyces* sp. NB-A13. *Bioorg. Chem.* **2019,** 82, 33-40.
5. Zhou, B.; Qin, L.; Ding, W.; Ma, Z. Cytotoxic indolocarbazoles alkaloids from the *Streptomyces* sp. A65. *Tetrahedron* **2018,** 74, (7), 726-730.
6. Wang, C.; Monger, A.; Wang, L.; Fu, P.; Piyachaturawat, P.; Chairoungdua, A.; Zhu, W. Precursor-directed generation of indolocarbazoles with topoisomerase IIα inhibitory activity. *Mar. Drugs* **2018,** 16, (5), 168.
7. Cui, T.; Lin, S.; Wang, Z.; Fu, P.; Wang, C.; Zhu, W. Cytotoxic indolocarbazoles from a marine-derived *Streptomyces* sp. OUCMDZ-5380. *Front. Microbiol.* **2022,** 13, 957473.
8. Zhang, W.; Ma, L.; Li, S.; Liu, Z.; Chen, Y.; Zhang, H.; Zhang, G.; Zhang, Q.; Tian, X.; Yuan, C.; Zhang, S.; Zhang, W.; Zhang, C. Indimicins A–E, bisindole alkaloids from the deep-sea-derived *Streptomyces* sp. SCSIO 03032. *J. Nat. Prod.* **2014,** 77, (8), 1887-1892.
9. Liu, Z.; Ma, L.; Zhang, L.; Zhang, W.; Zhu, Y.; Chen, Y.; Zhang, W.; Zhang, C. Functional characterization of the halogenase SpmH and discovery of new deschloro-tryptophan dimers. *Org. Biomol. Chem.* **2019,** 17, (5), 1053-1057.
10. Paulus, C.; Rebets, Y.; Tokovenko, B.; Nadmid, S.; Terekhova, L. P.; Myronovskyi, M.; Zotchev, S. B.; Ruckert, C.; Braig, S.; Zahler, S.; Kalinowski, J.; Luzhetskyy, A. New natural products identified by combined genomics-metabolomics profiling of marine *Streptomyces* sp. MP131-18. *Sci. Rep.* **2017,** 7, 42382.
11. Song, Y.; Yang, J.; Yu, J.; Li, J.; Yuan, J.; Wong, N. K.; Ju, J. Chlorinated *bis*-indole alkaloids from deep-sea derived *Streptomyces* sp. SCSIO 11791 with antibacterial and cytotoxic activities. *J. Antibiot. (Tokyo)* **2020,** 73, (8), 542-547.
12. Kim, S. H.; Ha, T. K.; Oh, W. K.; Shin, J.; Oh, D. C. Antiviral indolosesquiterpenoid xiamycins C-E from a halophilic actinomycete. *J. Nat. Prod.* **2016,** 79, (1), 51-8.

13. Park, J.; Cho, H. S.; Moon, D. H.; Lee, D.; Kal, Y.; Cha, S.; Lee, S. K.; Yoon, Y. J.; Oh, D.-C. Discovery of new indolosesquiterpenoids bearing a N-O linkage by overexpression of LuxR regulator in a marine bacterium *Streptomyces* sp. *Front. Mar. Sci.* **2023,** 10, 1140516.
14. Zhang, S.; Yang, Q.; Guo, L.; Zhang, Y.; Feng, L.; Zhou, L.; Yang, S.; Yao, Q.; Pescitelli, G.; Xie, Z. Isolation, structure elucidation and racemization of (+)- and (-)-pratensilins A-C: unprecedented spiro indolinone-naphthofuran alkaloids from a marine *Streptomyces* sp. *Chem. Commun. (Camb.)* **2017,** 53, (72), 10066-10069.
15. Guo, L.; Zhang, L.; Yang, Q.; Xu, B.; Fu, X.; Liu, M.; Li, Z.; Zhang, S.; Xie, Z. Antibacterial and cytotoxic bridged and ring cleavage angucyclinones from a marine *Streptomyces* sp. *Front. Chem.* **2020,** 8, 586.
16. Che, Q.; Qiao, L.; Han, X.; Liu, Y.; Wang, W.; Gu, Q.; Zhu, T.; Li, D. Anthranosides A-C, anthranilate derivatives from a sponge-derived *Streptomyces* sp. CMN-62. *Org. Lett.* **2018,** 20, (17), 5466-5469.
17. Newaz, A. W.; Yong, K.; Lian, X.; Zhang, Z. Streptoindoles A–D, novel antimicrobial indole alkaloids from the marine-associated actinomycete *Streptomyces* sp. ZZ1118. *Tetrahedron* **2022,** 104, 132598.
18. Zhang, X.; Li, Z.; Du, L.; Chlipala, G. E.; Lopez, P. C.; Zhang, W.; Sherman, D. H.; Li, S. Identification of an unexpected shunt pathway product provides new insights into tirandamycin biosynthesis. *Tetrahedron Lett.* **2016,** 57, (52), 5919-5923.
19. Cong, Z.; Huang, X.; Liu, Y.; Liu, Y.; Wang, P.; Liao, S.; Yang, B.; Zhou, X.; Huang, D.; Wang, J. Cytotoxic anthracycline and antibacterial tirandamycin analogues from a marine-derived *Streptomyces* sp. SCSIO 41399. *J. Antibiot. (Tokyo)* **2019,** 72, (1), 45-49.
20. Song, F.; Hu, J.; Zhang, X.; Xu, W.; Yang, J.; Li, S.; Xu, X. Unique cyclized thiolopyrrolones from the marine-derived *Streptomyces* sp. BTBU20218885. *Mar. Drugs* **2022,** 20, (3), 214.
21. Chung, B.; Hwang, J. Y.; Park, S. C.; Kwon, O. S.; Cho, E.; Lee, J.; Lee, H. S.; Oh, D. C.; Shin, J.; Oh, K. B. Inhibitory effects of nitrogenous metabolites from a marine-derived *Streptomyces bacillaris* on isocitrate lyase of *Candida albicans*. *Mar. Drugs* **2022,** 20, (2), 138.
22. Kim, M. C.; Lee, J. H.; Shin, B.; Subedi, L.; Cha, J. W.; Park, J. S.; Oh, D. C.; Kim, S. Y.; Kwon, H. C. Salinazinones A and B: Pyrrolidinyl-oxazinones from solar saltern-derived *Streptomyces* sp. KMF-004. *Org. Lett.* **2015,** 17, (20), 5024-7.
23. Zhang, Y. M.; Liu, B. L.; Zheng, X. H.; Huang, X. J.; Li, H. Y.; Zhang, Y.; Zhang, T. T.; Sun, D. Y.; Lin, B. R.; Zhou, G. X. Anandins A and B, two rare steroidal alkaloids from a marine *Streptomyces anandii* H41-59. *Mar. Drugs* **2017,** 15, (11), 355.
24. Lim, H. J.; An, J. S.; Bae, E. S.; Cho, E.; Hwang, S.; Nam, S. J.; Oh, K. B.; Lee, S. K.; Oh, D. C. Ligiamycins A and B, decalin-amino-maleimides from the co-culture of *Streptomyces* sp. and *Achromobacter* sp. Isolated from the marine wharf roach, *Ligia exotica*. *Mar. Drugs* **2022,** 20, (2), 83.
25. Han, Y.; Li, Y. Y.; Shen, Y.; Li, J.; Li, W. J.; Shen, Y. M. Oxoprothracarcin, a novel pyrrolo[1,4]benzodiazepine antibiotic from marine *Streptomyces* sp.

M10946. *Drug Discov. Ther.* **2013,** 7, (6), 243-7.
26. Çetinel Aksoy, S.; Küçüksolak, M.; Uze, A.; Bedir, E. Benzodiazepine derivatives from marine-derived *Streptomyces cacaoi* 14CM034. *Rec. Nat. Prod.* **2021,** 15, (6), 602-607.
27. Lu, C.; Li, Y.; Deng, J.; Li, S.; Shen, Y.; Wang, H.; Shen, Y. Hygrocins C-G, cytotoxic naphthoquinone ansamycins from gdmAI-disrupted *Streptomyces* sp. LZ35. *J. Nat. Prod.* **2013,** 76, (12), 2175-9.
28. Li, S.; Lu, C.; Ou, J.; Deng, J.; Shen, Y. Overexpression of hgc1 increases the production and diversity of hygrocins in *Streptomyces* sp. LZ35. *RSC Adv.* **2015,** 5, (102), 83843-83846.
29. Yi, W.; Newaz, A. W.; Yong, K.; Ma, M.; Lian, X. Y.; Zhang, Z. New Hygrocins K-U and streptophenylpropanamide A and bioactive compounds from the marine-associated *Streptomyces* sp. ZZ1956. *Antibiotics-Basel* **2022,** 11, (11), 1455.
30. Le, T. C.; Yang, I.; Yoon, Y. J.; Nam, S. J.; Fenical, W. Ansalactams B-D illustrate further biosynthetic plasticity within the ansamycin pathway. *Org. Lett.* **2016,** 18, (9), 2256-9.
31. Zhou, L. M.; Kong, F. D.; Xie, Q. Y.; Ma, Q. Y.; Hu, Z.; Zhao, Y. X.; Luo, D. Q. Divergolides T-W with apoptosis-inducing activity from the mangrove-derived actinomycete *Streptomyces* sp. KFD18. *Mar. Drugs* **2019,** 17, (4), 219.
32. Alvarez-Mico, X.; Jensen, P. R.; Fenical, W.; Hughes, C. C. Chlorizidine, a cytotoxic 5H-pyrrolo[2,1-a]isoindol-5-one-containing alkaloid from a marine *Streptomyces* sp. *Org. Lett.* **2013,** 15, (5), 988-991.
33. Heo, C. S.; Kang, J. S.; Kwon, J. H.; Anh, C. V.; Shin, H. J. Pyrrole-containing alkaloids from a marine-derived actinobacterium *Streptomyces zhaozhouensis* and their antimicrobial and cytotoxic activities. *Mar. Drugs* **2023,** 21, (3), 167.
34. Lian, X. Y.; Zhang, Z. Indanomycin-related antibiotics from marine *Streptomyces antibioticus* PTZ0016. *Nat. Prod. Res.* **2013,** 27, (23), 2161-7.
35. Zhou, B.; Huang, Y.; Zhang, H.; Li, J.; Ding, W. Nitricquinomycins A-C, uncommon naphthopyrolediones from the *Streptomyces* sp. ZS-A45. *Tetrahedron* **2019,** 75, (30), 3958-3961.
36. Song, F.; Yang, N.; Khalil, Z. G.; Salim, A. A.; Han, J.; Bernhardt, P. V.; Lin, R.; Xu, X.; Capon, R. J. Bhimamycin J, a rare benzo[*f*]isoindole-dione alkaloid from the marine-derived actinomycete *Streptomyces* sp. MS180069. *Chem. Biodivers.* **2021,** 18, (11), e2100674.
37. Ko, K.; Kim, S. H.; Park, S.; Han, H. S.; Lee, J. K.; Cha, J. W.; Hwang, S.; Choi, K. Y.; Song, Y. J.; Nam, S. J.; Shin, J.; Nam, S. I.; Kwon, H. C.; Park, J. S.; Oh, D. C. Discovery and photoisomerization of new pyrrolosesquiterpenoids glaciapyrroles D and E, from deep-sea sediment *Streptomyces* sp. *Mar. Drugs* **2022,** 20, (5), 281.
38. Liu, Y.; Ding, L.; Deng, Y.; Wang, X.; Cui, W.; He, S. Feature-based molecular networking-guided discovery of siderophores from a marine mesophotic

zone *Axinellida* sponge-associated actinomycete *Streptomyces diastaticus* NBU2966. *Phytochemistry* **2022,** 196, 113078.
39. Han, X.; Liu, Z.; Zhang, Z.; Zhang, X.; Zhu, T.; Gu, Q.; Li, W.; Che, Q.; Li, D. Geranylpyrrol A and piericidin F from *Streptomyces* sp. CHQ-64 Δ*rdmF*. *J. Nat. Prod.* **2017,** 80, (5), 1684-1687.
40. Zhou, X.; Liang, Z.; Li, K.; Fang, W.; Tian, Y.; Luo, X.; Chen, Y.; Zhan, Z.; Zhang, T.; Liao, S.; Liu, S.; Liu, Y.; Fenical, W.; Tang, L. Exploring the natural piericidins as anti-renal cell carcinoma agents targeting peroxiredoxin 1. *J. Med. Chem.* **2019,** 62, (15), 7058-7069.
41. Peng, J.; Zhang, Q.; Jiang, X.; Ma, L.; Long, T.; Cheng, Z.; Zhang, C.; Zhu, Y. New piericidin derivatives from the marine-derived *Streptomyces* sp. SCSIO 40063 with cytotoxic activity. *Nat. Prod. Res.* **2022,** 36, (10), 2458-2464.
42. Hassan, H. M.; Boonlarppradab, C.; Fenical, W. Actinoquinolines A and B, anti-inflammatory quinoline alkaloids from a marine-derived *Streptomyces* sp., strain CNP975. *J. Antibiot. (Tokyo)* **2016,** 69, (7), 511-4.
43. Ortiz-Lopez, F. J.; Alcalde, E.; Sarmiento-Vizcaino, A.; Diaz, C.; Cautain, B.; Garcia, L. A.; Blanco, G.; Reyes, F. New 3-hydroxyquinaldic acid derivatives from cultures of the marine derived actinomycete *Streptomyces cyaneofuscatus* M-157. *Mar. Drugs* **2018,** 16, (10), 371.
44. Mullowney, M. W.; E, O. h.; Shaikh, A.; Wei, X.; Tanouye, U.; Santarsiero, B. D.; Burdette, J. E.; Murphy, B. T. Diazaquinomycins E-G, novel diaza-anthracene analogs from a marine-derived *Streptomyces* sp. *Mar. Drugs* **2014,** 12, (6), 3574-3586.
45. Cheng, C.; Othman, E. M.; Reimer, A.; Grüne, M.; Kozjak-Pavlovic, V.; Stopper, H.; Hentschel, U.; Abdelmohsen, U. R. Ageloline A, new antioxidant and antichlamydial quinolone from the marine sponge-derived bacterium *Streptomyces* sp. SBT345. *Tetrahedron Lett.* **2016,** 57, (25), 2786-2789.
46. Shaaban, M.; Shaaban, K. A.; Kelter, G.; Fiebig, H. H.; Laatsch, H. Mansouramycins E–G, cytotoxic isoquinolinequinones from marine *Streptomycetes*. *Mar. Drugs* **2021,** 19, (12), 715.
47. Yang, C. L.; Wang, Y. S.; Liu, C. L.; Zeng, Y. J.; Cheng, P.; Jiao, R. H.; Bao, S. X.; Huang, H. Q.; Tan, R. X.; Ge, H. M. Strepchazolins A and B: Two new alkaloids from a marine *Streptomyces chartreusis* NA02069. *Mar. Drugs* **2017,** 15, (8), 244.
48. Zhang, D.; Yi, W.; Ge, H.; Zhang, Z.; Wu, B. Bioactive streptoglutarimides A-J from the marine-derived *Streptomyces* sp. ZZ741. *J. Nat. Prod.* **2019,** 82, (10), 2800-2808.
49. Lee, D. S.; Yoon, C. S.; Jung, Y. T.; Yoon, J. H.; Kim, Y. C.; Oh, H. Marine-derived secondary metabolite, griseusrazin A, suppresses inflammation through heme oxygenase-1 induction in activated RAW264.7 macrophages. *J. Nat. Prod.* **2016,** 79, (4), 1105-11.
50. Shaala, L. A.; Youssef, D. T.; Badr, J. M.; Harakeh, S. M. Bioactive 2(1*H*)-pyrazinones and diketopiperazine alkaloids from a tunicate-derived actinomycete *Streptomyces* sp. *Molecules* **2016,** 21, (9), 1116.
51. Chen, M.; Chai, W.; Zhu, R.; Song, T.; Zhang, Z.; Lian, X. Streptopyrazinones A-D, rare metabolites from marine-derived *Streptomyces* sp. ZZ446.

Tetrahedron **2018,** 74, (16), 2100-2106.
52. Kim, S.; Hillman, P. F.; Lee, J. Y.; Lee, J.; Lee, J.; Cha, S.-S.; Oh, D.-C.; Nam, S.-J.; Fenical, W. Actinopolymorphols E and F, pyrazine alkaloids from a marine sediment-derived bacterium *Streptomyces* sp. *J. Antibiot. (Tokyo)* **2022,** 75, (11), 619-625.
53. Ou, Y. X.; Huang, J. F.; Li, X. M.; Kang, Q. J.; Pan, Y. T. Three new 2,5-diketopiperazines from the fish intestinal *Streptomyces* sp. MNU FJ-36. *Nat. Prod. Res.* **2016,** 30, (15), 1771-5.
54. Yi, W.; Qin, L.; Lian, X. Y.; Zhang, Z. New antifungal metabolites from the Mariana Trench sediment-associated actinomycete *Streptomyces* sp. SY1965. *Mar. Drugs* **2020,** 18, (8), 385.
55. Buedenbender, L.; Grkovic, T.; Duffy, S.; Kurtböke, D. I.; Avery, V. M.; Carroll, A. R. Naseseazine C, a new anti-plasmodial dimeric diketopiperazine from a marine sediment derived *Streptomyces* sp. *Tetrahedron Lett.* **2016,** 57, (52), 5893-5895.
56. Shaala, L. A.; Youssef, D. T. A.; Badr, J. M.; Harakeh, S. M.; Genta-Jouve, G. Bioactive diketopiperazines and nucleoside derivatives from a sponge-derived *Streptomyces* species. *Mar. Drugs* **2019,** 17, (10), 584.
57. Chen, S.; Zhang, D.; Chen, M.; Zhang, Z.; Lian, X. Y. A rare diketopiperazine glycoside from marine-sourced *Streptomyces* sp. ZZ446. *Nat. Prod. Res.* **2020,** 34, (7), 1046-1050.
58. Ren, J.; Liu, D.; Tian, L.; Wei, Y.; Proksch, P.; Zeng, J.; Lin, W. Venezuelines A-G, new phenoxazine-based alkaloids and aminophenols from *Streptomyces venezuelae* and the regulation of gene target Nur77. *Bioorg. Med. Chem. Lett.* **2013,** 23, (1), 301-4.
59. Abdelfattah, M. S. A new bioactive aminophenoxazinone alkaloid from a marine-derived actinomycete. *Nat. Prod. Res.* **2013,** 27, (22), 2126-31.
60. Kunz, A. L.; Labes, A.; Wiese, J.; Bruhn, T.; Bringmann, G.; Imhoff, J. F. Nature's lab for derivatization: new and revised structures of a variety of streptophenazines produced by a sponge-derived *Streptomyces* strain. *Mar. Drugs* **2014**, 12, (4), 1699-1714.
61. Cheng, C.; Othman, E. M.; Fekete, A.; Krischke, M. Strepoxazine A, a new cytotoxic phenoxazin from the marine sponge-derived bacterium *Streptomyces* sp. SBT345. *Tetrahedron Lett.* **2016,** 57, (37), 4196-4199.
62. Wang, Q.; Zhang, Y.; Wang, M.; Tan, Y.; Hu, X.; He, H.; Xiao, C.; You, X.; Wang, Y.; Gan, M. Neo-actinomycins A and B, natural actinomycins bearing the 5H-oxazolo[4,5-b]phenoxazine chromophore, from the marine-derived *Streptomyces* sp. IMB094. *Sci. Rep.* **2017,** 7, (1), 3591.
63. Zhao, W.; Wang, G.; Guo, L.; Wang, J.; Jing, C.; Liu, B.; Zhao, F.; Zhang, S.; Xie, Z. Asp-containing actinomycin and tetracyclic chromophoric analogues from the *Streptomyces* sp. strain S22. *Org. Biomol. Chem.* **2023,** 21, (8), 1737-1743.
64. Jiang, Y.-J.; Li, J.-Q.; Zhang, H.-J.; Ding, W.-J.; Ma, Z.-J. Cyclizidine-type alkaloids from *Streptomyces* sp. HNA39. *J. Nat. Prod.* **2018,** 81, 394-399.
65. Cheng, X. W.; Li, J. Q.; Jiang, Y. J.; Liu, H. Z.; Huo, C. A new indolizinium alkaloid from marine-derived *Streptomyces* sp. HNA39. *J. Asian Nat. Prod.*

Res. **2021,** 23, (9), 913-918.
66. Shi, Y.; Pan, C.; Auckloo, B. N.; Chen, X.; Chen, C. A.; Wang, K.; Wu, X.; Ye, Y.; Wu, B. Stress-driven discovery of a cryptic antibiotic produced by Streptomyces sp. WU20 from Kueishantao hydrothermal vent with an integrated metabolomics strategy. *Appl. Microbiol. Biotechnol.* **2017**, 101, (4), 1395-1408.
67. Zhang, X.; Chen, L.; Chai, W.; Lian, X.-Y.; Zhang, Z. A unique indolizinium alkaloid streptopertusacin A and bioactive bafilomycins from marine-derived *Streptomyces* sp. HZP-2216E. *Phytochemistry* **2017**, 144, 119-126.
68. Fu, P.; MacMillan, J. B. Spithioneines A and B, two new bohemamine derivatives possessing ergothioneine moiety from a marine-derived *Streptomyces spinoverrucosus*. *Org. Lett.* **2015,** 17, (12), 3046-9.
69. Fu, P.; La, S.; MacMillan, J. B. 1,3-oxazin-6-one derivatives and bohemamine-type pyrrolizidine alkaloids from a marine-derived *Streptomyces spinoverrucosus*. *J. Nat. Prod.* **2016,** 79, (3), 455-62.
70. Fu, P.; Legako, A.; La, S.; MacMillan, J. B. Discovery, Characterization, and Analogue Synthesis of Bohemamine Dimers Generated by Non-enzymatic Biosynthesis. *Chemistry* **2016,** 22, (10), 3491-3495.
71. Fu, P.; Johnson, M.; Chen, H.; Posner, B. A.; MacMillan, J. B. Carpatamides A-C, cytotoxic arylamine derivatives from a marine-derived *Streptomyces* sp. *J. Nat. Prod.* **2014,** 77, (5), 1245-8.
72. Zhang, W.; Che, Q.; Tan, H.; Qi, X.; Li, J.; Li, D.; Gu, Q.; Zhu, T.; Liu, M. Marine *Streptomyces* sp. derived antimycin analogues suppress HeLa cells via depletion HPV E6/E7 mediated by ROS-dependent ubiquitin-proteasome system. *Sci. Rep.* **2017,** 7, 42180.
73. Hu, C.; Zhou, S. W.; Chen, F.; Zheng, X. H.; Shen, H. F.; Lin, B. R.; Zhou, G. X. Neoantimycins A and B, two unusual benzamido nine-membered dilactones from marine-derived *Streptomyces antibioticus* H12-15. *Molecules* **2017,** 22, (4), 557.
74. Chen, L.; Chai, W.; Wang, W.; Song, T.; Lian, X. Y.; Zhang, Z. Cytotoxic bagremycins from mangrove-derived *Streptomyces* sp. Q22. *J. Nat. Prod.* **2017,** 80, (5), 1450-1456.
75. Zhang, D.; Shu, C.; Lian, X.; Zhang, Z. New antibacterial bagremycins F and G from the marine-derived *Streptomyces* sp. ZZ745. *Mar. Drugs* **2018,** 16, (9), 330.
76. Hamed, A.; Abdel-Razek, A. S.; Frese, M.; Wibberg, D.; El-Haddad, A. F.; Ibrahim, T. M. A.; Kalinowski, J.; Sewald, N.; Shaaban, M. N-Acetylborrelidin B: a new bioactive metabolite from *Streptomyces mutabilis* sp. MII. *Z. Naturforsch. C* **2018,** 73, (1-2), 49-57.
77. Lacret, R.; Oves-Costales, D.; Gómez, C.; Díaz, C.; de la Cruz, M.; Pérez-Victoria, I.; Vicente, F.; Genilloud, O.; Reyes, F. New ikarugamycin derivatives with antifungal and antibacterial properties from *Streptomyces zhaozhouensis*. *Mar. Drugs* **2014,** 13, (1), 128-140.

78. Saha, S.; Zhang, W.; Zhang, G.; Zhu, Y.; Chen, Y.; Liu, W.; Yuan, C.; Zhang, Q.; Zhang, H.; Zhang, L.; Zhang, W.; Zhang, C. Activation and characterization of a cryptic gene cluster reveals a cyclization cascade for polycyclic tetramate macrolactams. *Chem. Sci.* **2017,** 8, (2), 1607-1612.
79. Fukudaa, T.; Takahashia, M.; Kasaib, H.; Nagaia, K.; Tomodaa, H. Chlokamycin, a new chloride from the marine-derived *Streptomyces* sp. MA2-12. *Nat. Prod. Commun.* **2017,** 12, (8), 1223-1226.
80. Liu, W.; Zhang, W.; Jin, H.; Zhang, Q.; Chen, Y.; Jiang, X.; Zhang, G.; Zhang, L.; Zhang, W.; She, Z.; Zhang, C. Genome mining of marine-derived *Streptomyces* sp. SCSIO 40010 leads to cytotoxic new polycyclic tetramate macrolactams. *Mar. Drugs* **2019,** 17, (12), 663.
81. Kawahara, T.; Fujiwara, T.; Kagaya, N.; Shin-Ya, K. JBIR-150, a novel 20-membered polyene macrolactam from marine-derived *Streptomyces* sp. OPMA00071. *J. Antibiot. (Tokyo)* **2018,** 71, (3), 390-392.
82. Shin, Y. H.; Im, J. H.; Kang, I.; Kim, E.; Jang, S. C.; Cho, E.; Shin, D.; Hwang, S.; Du, Y. E.; Huynh, T. H.; Ko, K.; Ko, Y. J.; Nam, S. J.; Awakawa, T.; Lee, J.; Hong, S.; Abe, I.; Moore, B. S.; Fenical, W.; Yoon, Y. J.; Cho, J. C.; Lee, S. K.; Oh, K. B.; Oh, D. C. Genomic and spectroscopic signature-based discovery of natural macrolactams. *J. Am. Chem. Soc.* **2023,** 145, (3), 1886-1896.
83. Shen, J.; Wang, J.; Chen, H.; Wang, Y.; Zhu, W.; Fu, P. Cyclamenols E and F, two diastereoisomeric bicyclic macrolactams with a cyclopentane moiety from an Antarctic *Streptomyces* species. *Org. Chem. Front.* **2020,** 7, (2), 310-317.
84. Yang, F.; Sang, M.; Lu, J. R.; Zhao, H. M.; Zou, Y.; Wu, W.; Yu, Y.; Liu, Y. W.; Ma, W.; Zhang, Y.; Zhang, W.; Lin, H. W. Somalactams A-D: Anti-inflammatory macrolide lactams with unique ring systems from an Arctic actinomycete strain. *Angew. Chem. Int. Ed. Engl.* **2023,** 62, (18), e202218085.
85. Hu, Y.; Wang, M.; Wu, C.; Tan, Y.; Li, J.; Hao, X.; Duan, Y.; Guan, Y.; Shang, X.; Wang, Y.; Xiao, C.; Gan, M. Identification and proposed relative and absolute configurations of niphimycins C-E from the marine-derived *Streptomyces* sp. IMB7-145 by genomic analysis. *J. Nat. Prod.* **2018,** 81, (1), 178-187.
86. Kim, D.; Lee, E. J.; Lee, J.; Leutou, A. S.; Shin, Y. H.; Choi, B.; Hwang, J. S.; Hahn, D.; Choi, H.; Chin, J.; Cho, S. J.; Hong, Y. D.; Ko, J.; Seong, C. N.; Maloney, K. N.; Oh, D. C.; Yang, I.; Hwang, H.; Nam, S. J. Antartin, a cytotoxic zizaane-type sesquiterpenoid from a *Streptomyces* sp. isolated from an Antarctic marine sediment. *Mar. Drugs* **2018,** 16, (4), 130.
87. Liu, W.; Ma, L.; Zhang, L.; Chen, Y.; Zhang, Q.; Zhang, H.; Zhang, W.; Zhang, C.; Zhang, W. Two new phenylhydrazone derivatives from the Pearl River Estuary sediment-derived *Streptomyces* sp. SCSIO 40020. *Mar. Drugs* **2022,** 20, (7), 449.
